# Chemical Incorporation of Chain-Terminating Nucleoside Analogs as 3′-Blocking DNA Damage and Their Removal by Human ERCC1-XPF Endonuclease

**DOI:** 10.3390/molecules21060766

**Published:** 2016-06-11

**Authors:** Junpei Yamamoto, Chiaki Takahata, Isao Kuraoka, Kouji Hirota, Shigenori Iwai

**Affiliations:** 1 Division of Chemistry, Graduate School of Engineering Science, Osaka University, 1-3 Machikaneyama, Toyonaka, Osaka 560-8531, Japan; takahata@bio.chem.es.osaka-u.ac.jp (C.T.); kuraoka@chem.es.osaka-u.ac.jp (I.K.); iwai@chem.es.osaka-u.ac.jp (S.I.); 2 Department of Chemistry, Tokyo Metropolitan University, 1-1 Minami-Ohsawa, Hachi-Ohji, Tokyo 192-0397, Japan; khirota@tmu.ac.jp

**Keywords:** DNA damage, DNA repair, antiviral agents, solid-phase synthesis, nucleotide excision repair

## Abstract

Nucleoside/nucleotide analogs that lack the 3′-hydroxy group are widely utilized for HIV therapy. These chain-terminating nucleoside analogs (CTNAs) block DNA synthesis after their incorporation into growing DNA, leading to the antiviral effects. However, they are also considered to be DNA damaging agents, and tyrosyl-DNA phosphodiesterase 1, a DNA repair enzyme, is reportedly able to remove such CTNA-modifications of DNA. Here, we have synthesized phosphoramidite building blocks of representative CTNAs, such as acyclovir, abacavir, carbovir, and lamivudine, and oligonucleotides with the 3′-CTNAs were successfully synthesized on solid supports. Using the chemically synthesized oligonucleotides, we investigated the excision of the 3′-CTNAs in DNA by the human excision repair cross complementing protein 1-xeroderma pigmentosum group F (ERCC1-XPF) endonuclease, which is one of the main components of the nucleotide excision repair pathway. A biochemical analysis demonstrated that the ERCC1-XPF endonuclease cleaved 2–7 nt upstream from the 3′-blocking CTNAs, and that DNA synthesis by the Klenow fragment was resumed after the removal of the CTNAs, suggesting that ERCC1-XPF participates in the repair of the CTNA-induced DNA damage.

## 1. Introduction

**Figure 1:**
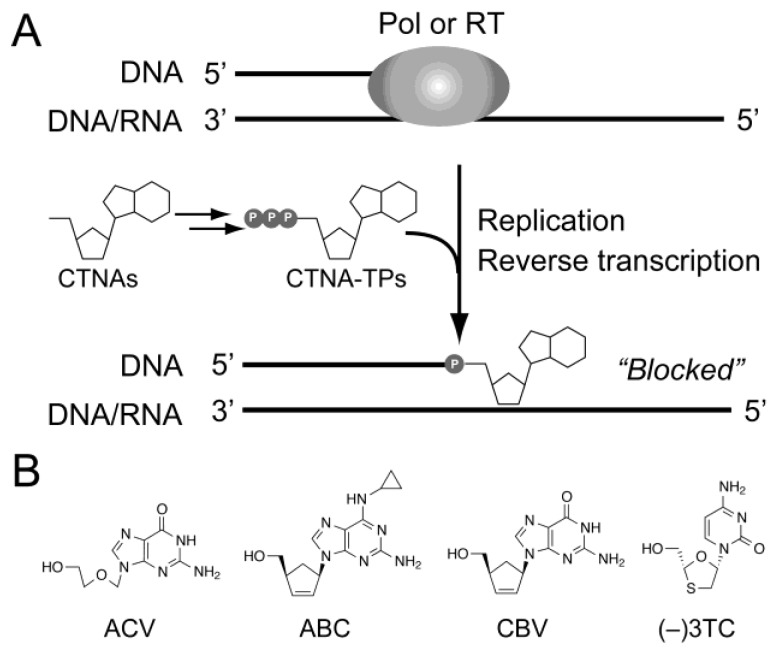
Chain-terminating nucleoside analogs (CTNAs). (**A**) Mechanism of the antiviral effect by the CTNAs; (**B**) The structures of the CTNAs used in this study.

Nucleoside/nucleotide analogs lacking their 3′-hydroxyl group, such as 3′-azidothymidine (AZT), function as efficient antiviral drugs. Their common therapeutic target is DNA synthesis by DNA polymerases or reverse transcriptases (RTs) [1]. They are first metabolized into the corresponding 5′-monophosphate derivatives by cellular nucleoside kinases, and then phosphorylated for conversion to the triphosphate forms. The drug triphosphates either inhibit the correct binding of the intact nucleotides to polymerases/RTs or are incorporated into the growing viral DNA. The chain elongation in reverse transcription and DNA replication is halted once the drug triphosphates have been incorporated (Figure 1A), due to the inability to form the 5′–3′ phosphodiester bonds during DNA synthesis. These chain-terminating nucleoside analogs (CTNAs) are widely utilized for HIV therapy [2].

However, since they can potentially block DNA synthesis, the toxic side effects of CTNAs in the host’s mitochondria have presented clinical problems [3]. DNA polymerase γ (Polγ) is the sole enzyme for mtDNA replication, and it can bind the drug triphosphates and incorporate them into mtDNA [4]. Polγ has an intrinsic proofreading 3′–5′ exonuclease activity that can remove the incorporated CTNAs [5]. Lee *et al.* reported that the clinical toxicities of CTNAs correlated well with their potential toxicities, which were calculated from the *in vitro* kinetic rates of CTNA incorporation and CTNA removal by Polγ [6]. Indeed, the 3′–5′ exonuclease activity of Polγ and those of the replicative DNA polymerases [7] can excise the CTNAs in DNA. However, their rates are relatively slow as compared to that for the removal of normal nucleotides, suggesting that the removal of the 3′-terminal CTNAs may be accomplished by the proofreading function, as well as other processes.

Human tyrosyl-DNA phosphodiesterase 1 (TDP1) is the DNA repair enzyme responsible for the removal of 3′-phosphotyrosyl DNA, which is formed by the enzymatic digestion of a covalent complex between DNA and topoisomerase 1 [8]. Recently, the human TDP1, encoded by the nuclear gene, was reported to be localized to mitochondria in addition to the nucleus for oxidative DNA damage repair [9]. Additionally, this enzyme exhibited the activity to cleave the 3′-terminal CTNAs, and Tdp1-deficient DT40 cells displayed hypersensitivity upon CTNA treatment [10]. In fact, cells from adult T-cell leukemia (ATL), which is caused by the infection of human T-cell leukemia virus-1, lack TDP1-dependent DNA repair activity, and recently it was reported that abacavir, one of the CTNAs developed for the anti-HIV agent, can kill ATL cells selectively [11]. This is considered to be due to the impaired DNA repair of the abacavir-induced DNA damage, and thus repair of the CTNA-induced DNA damage could be a new potential target for cancer therapy. So far, repair of the CTNA-incorporated DNA has been shown only for TDP1, but other DNA repair enzymes would potentially be involved as well.

In order to investigate the repair of CTNA-induced DNA damage *in vitro*, oligonucleotides containing 3′-CTNAs are required. For the preparation of the substrates containing the 3′-CTNAs, the 5′-triphosphate derivatives of the CTNAs are incorporated at the 3′-termini of the oligonucleotides by DNA polymerases [4,5,7,10]. This preparation method is advantageous for the electrophoretic analysis of CTNA incorporation and removal. However, a serious drawback is the limited amount of substrates available for structural and biophysical analyses. To facilitate research on CTNA-induced DNA damage, automated DNA synthesis using phosphoramidite chemistry will be useful for the preparation of CTNA-modified oligonucleotides.

**Scheme 1:**
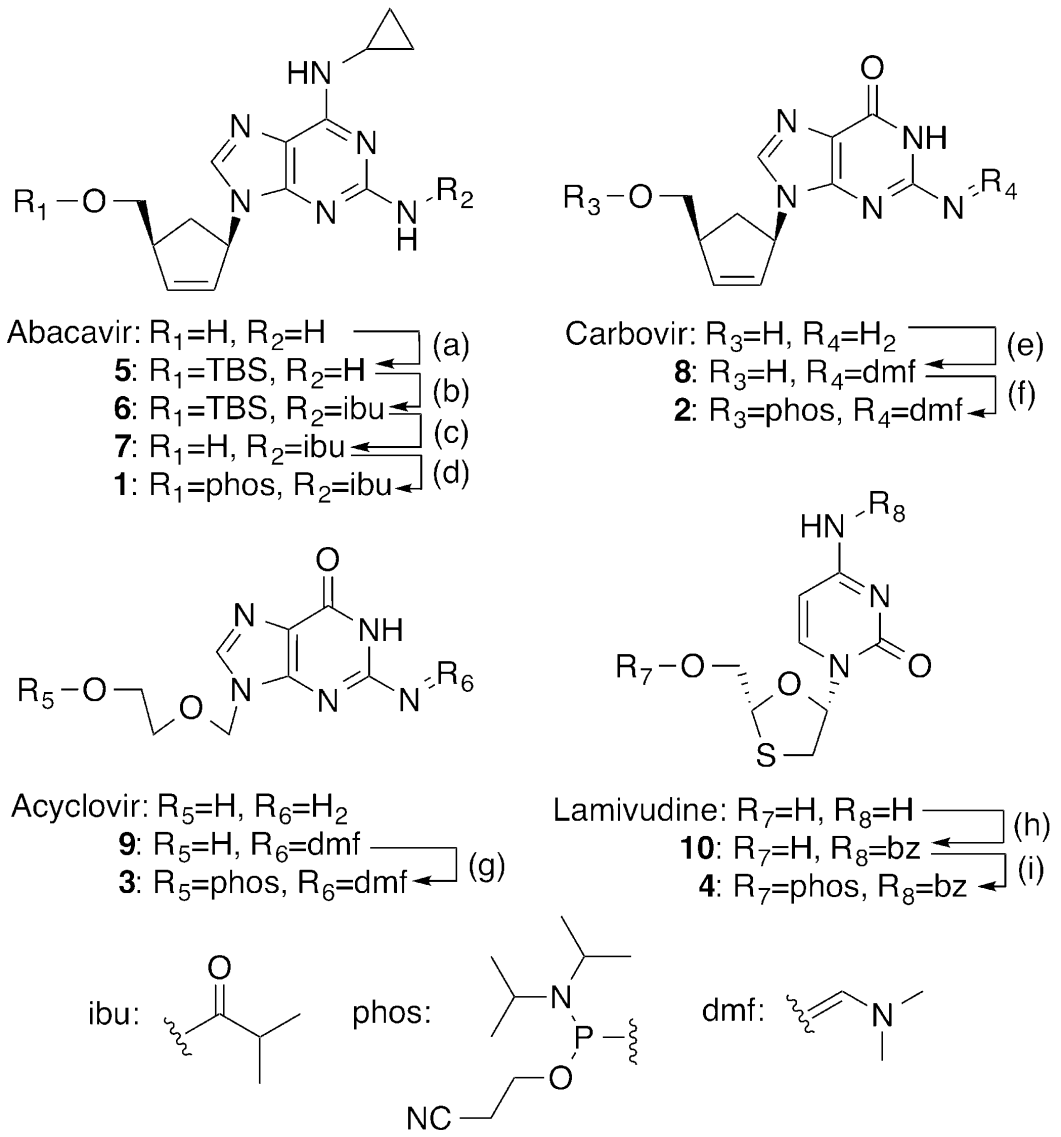
Synthesis of the phosphoramidites of the chain-terminating nucleoside analogs. *Reagents and conditions*: (a) TBSCl, imidazole/pyridine, r.t., 20 h, 93%; (b) isobutyryl chloride/pyridine, r.t., 1 h, 88%; (c) TEA·3HF/THF, r.t., overnight, 89%; (d) DIPEA, 2-cyanoethyl diisopropylchloro-phosphoramidite/THF, r.t., 30 min, 56%; (e) DMF(OCH_3_)_2_/DMF, r.t., overnight, 95%; (f) DIPEA, 2-cyanoethyl diisopropylchlorophosphoramidite/acetonitrile, r.t., 30 min, 56%; (g) DIPEA, 2-cyanoethyl diisopropylchlorophosphoramidite/THF and DMF, r.t., 30 min, 25%; (h) benzoic anhydride/ethanol, 70 °C, 2.5 h, 80%; and (i) DIPEA, 2-cyanoethyl diisopropylchlorophosphoramidite/THF, r.t., 40 min, 71%. Abbreviations: TBS = *tert*-butyldimethylsilyl; DIPEA = diisopropylethylamine; bz = benzoyl; ibu = isobutyryl; dmf = *N,N*-dimethylformamidyl; phos = 2-cyanoethyl-*N,N*-diisopropylphosphoramidyl.

Here, four representative CTNAs used as antiviral reagents, abacavir, carbovir (the active form of abacavir), acyclovir, and lamivudine, which can potentially be incorporated into DNA (Figure 1B), were derivatized into the corresponding phosphoramidite building blocks **1**–**4** (Scheme 1), and were then attached to the 3′-termini of oligonucleotides on a solid support. Using the CTNA-oligonucleotides, the 3′-CTNA removal by human excision repair cross complementing protein 1–xeroderma pigmentosum group F (ERCC1-XPF) complex, which is a structure-specific endonuclease involved in the nucleotide excision repair (NER) pathway, was investigated. The results show that ERCC1-XPF can excise the 3′-CTNAs and reproduce the extendable 3′-OH.

## 2. Results

### 2.1. Synthesis of 5′-Phosphoramidites of CTNAs

Abacavir (ABC) is a prodrug for HIV therapy approved by the FDA in 1998, and the cyclopropylmethylamino group is reportedly hydrolyzed by cellular deaminases to form its active structure, carbovir (CBV) [12]. Toward the synthesis of the ABC phosphoramidite **1**, the hydroxyl group was protected with a *tert*-butyldimethylsilyl (TBS) group, followed by the protection of the amino group at C2 with an isobutyryl group (Scheme 1). Compound **6** was derivatized into compound **1**, after the removal of the TBS group. CBV was synthesized from optically-pure (1*R*)-(−)-2-azabicyclo[2.2.1]hept-5-en-3-one, according to the previous reports [13,14]. The C2 amino function of CBV was protected with the *N,N*-dimethylformamidyl (dmf) group to yield compound **8**, which was finally phosphitylated to yield the CBV phosphoramidite **2**.

Acyclovir (ACV) is primarily used for the treatment of patients with herpes simplex virus infections [15]. The ACV phosphoramidite **3** was synthesized via compound **9**, in which the C2 amino group was protected with the dmf group [16]. However, *N*^2^-dmf ACV **9** was minimally soluble in organic solvents, and even after phosphitylation, compound **3** partitioned into both the organic and water layers, leading to the low isolation yield. Lamivudine (2’,3′-dideoxy-3′-thiacytidine, 3TC), is a characteristic drug for HIV therapy because its enantiomeric d- and l-forms have different clinical aspects, and the d-form ((+)3TC), which has the same stereochemistry as the natural nucleosides, is reportedly much more toxic than the l-form ((−)3TC) [17]. Consequently, the structural definition of 3TC is very important, and here we used (−)3TC as the starting material. The C4 amino group of (−)3TC was protected with a benzoyl group by a treatment with benzoic anhydride. The *N*^4^-benzoyl (−)3TC, **10** was then phosphorylated to yield compound **4**. Note that the same procedure is applicable to the synthesis of the (+)3TC phosphoramidite.

### 2.2. Chemical Synthesis of Oligonucleotides Containing CTNAs in the 5′ → 3′ Direction

By using the CTNA phosphoramidites **1**–**4** and the commercially-available nucleoside 5′-phosphoramidites, 19-mer oligonucleotides, d(TCCGTTGAAGCCTGCTTT)X, where X represents the CTNAs, were synthesized in the 5′ → 3′ direction. The synthesized oligonucleotides were deprotected by a treatment with ammonia water at 55 °C for 5 h. The protecting groups in the oligonucleotides containing ACV, CBV, and (−)3TC were removed successfully by this treatment, while prolonged treatment with hot aqueous ammonia (24 h in total) was required for the ABC-oligonucleotide to remove the isobutyryl group on ABC. After HPLC purification, the synthesized oligonucleotides were characterized by mass spectrometry (Table S1 and Figures S1–S4).

### 2.3. Primer Extension from the CTNA-Blocking Termini by Klenow Fragments

**Figure 2:**
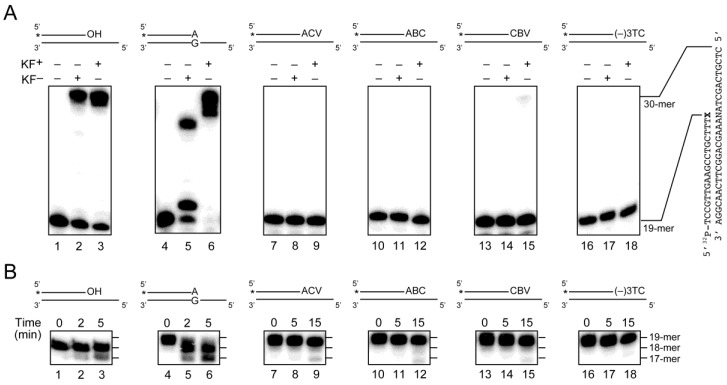
Primer extension from the CTNA-blocked termini by the Klenow fragment of *Escherichia coli* DNA polymerase I, with or without its proofreading 3′–5′ exonuclease activity (KF^+^ or KF^−^, respectively, from Takara Bio, Inc., Shiga, Japan), in the (**A**) presence or (**B**) absence of dNTPs. (**A**) The ^32^P-labeled oligonucleotides, ^32^P-d(TCCGTTGAAGCCTGCTTT)X, where X represents no added nucleoside (OH, lanes 1–3), 2’-deoxyadenosine (lanes 4–6), acyclovir (ACV, lanes 7–9), abacavir (ABC, lanes 10–12), carbovir (CBV, lanes 13–15) or lamivudine ((−)3TC, lanes 16–18), were hybridized with their complementary strands, d(CTCGTCAGCTANAAAGCAGGCTTCAACGGA), where N represents A (for ABC and an oligonucleotide without CTNAs), G (for A and (−)3TC) or C (for ACV and CBV). Each substrate was incubated at 37 °C for 10 min, in the absence (lanes 1, 4, 7, 10, 13 and 16) or presence of KF^−^ (0.1 unit, lanes 2, 5, 8, 11, 14 and 17) or KF^+^ (0.1 unit, lanes 3, 6, 9, 12, 15 and 18), in 10 mM Tris-HCl buffer (pH 7.9) containing 50 mM NaCl, 10 mM MgCl_2_, 10 mM DTT and 100 µM dNTPs; (**B**) The ^32^P-labeled substrates were incubated with KF^+^ at 37 °C for the indicated incubation time, in the same reaction buffer without dNTPs.

By using the synthesized oligonucleotides containing CTNAs, the exonucleolytic removal of the CTNAs by DNA polymerases was assessed. These oligonucleotides were labeled with ^32^P, and the removal of the 3′-CTNAs followed by the resumption of DNA synthesis by the Klenow fragment (KF) of *Escherichia coli* DNA polymerase I, with and without exonuclease activity (KF^+^ and KF^−^, respectively), was analyzed. As a positive control for the proofreading activity of KF^+^, an artificial A·G mismatched substrate was also prepared. As shown in Figure 2A, all of the CTNAs blocked chain elongation by KF^−^ (lanes 8, 11, 14 and 17). KF^−^ extended the mismatched substrate (lane 5), but the fully-extended product was not observed. In the presence of KF^+^, a slight amount (3.2%) of the fully-extended product, which is supposed to be obtained after the removal of the terminal CTNAs, was observed only for the CBV-modified substrate (lane 15), while KF^+^ almost completely removed the terminal A·G mismatch and resumed the DNA synthesis under the same reaction conditions (lane 6, 97% yield for the fully-extended product). To further confirm the resistance of the CTNAs to the exonuclease activity, the substrates were incubated with KF^+^ in the absence of dNTPs (Figure 2B). Although the A·G mismatched substrate was digested within 5 min (lane 6), the CTNA-containing substrates were not cleaved as efficiently as the mismatched substrate (lanes 8, 11, 14 and 17), and even upon longer incubation (15 min), only slight amounts of the digested products were observed (lanes 9, 12, 15 and 18). These results demonstrated that the terminal CTNA modifications introduced by phosphoramidite chemistry worked as chain terminators.

### 2.4. Removal of 3′-CTNAs by ERCC1-XPF Endonuclease in Vitro

Next, the removal of the 3′-CTNAs by ERCC1-XPF was investigated. ERCC1-XPF, the human homolog of yeast Rad10-Rad1 [18], is a structure-specific endonuclease that makes a 5′-incision in the damaged DNA strand during NER, and its deficiency causes an inherent genetic disease, xeroderma pigmentosum [19]. ERCC1-XPF recognizes a replication fork-like DNA structure, and preferentially cleaves the stem 2–8 nt upstream from the junction to produce the 3′-OH terminus [20]. In addition to the fork-like structure, ERCC1-XPF can also remove the 3′-blockage of single-stranded breaks with an α,β-unsaturated aldehyde [21] and phosphoglycolates [22]. Interestingly, ERCC1-XPF has been implicated in the removal of the 3′-phosphotyrosyl DNA, as an alternative to the TDP1-dependent pathway [23]. These previous investigations suggested that the 3′-blocking CTNAs, which are not processed by the exonucleolytic proofreading activity of KF, are expected to be recognized and removed by ERCC1-XPF in a similar manner.

**Figure 3:**
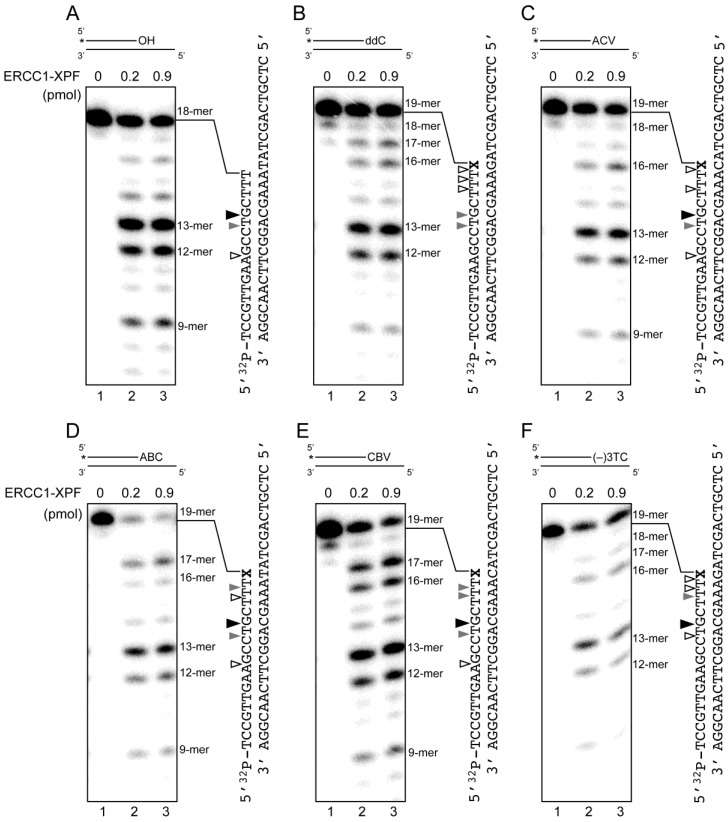
Removal of the CTNAs attached to the 3′-termini of oligonucleotides by human ERCC1-XPF endonuclease. The ^32^P-labeled substrates (400 fmol) containing (**A**) 3′-OH; (**B**) ddC; (**C**) ACV; (**D**) ABC; (**E**) CBV; and (**F**) (−)3TC were treated with increasing amounts of ERCC1-XPF (0, 230 and 920 fmol for lanes 1, 2 and 3, respectively) at 30 °C for 90 min, in 50 mM Tris-HCl buffer (pH 8.0) containing 0.5 mM MnCl_2_, 0.5 mM DTT and 0.1 mg·mL^−1^ bovine serum albumin (BSA). The cleaved products in lanes 3 were quantified, and the remarkable cleavage sites are indicated by black, gray and open triangles, depending on the yield of each product (>20%, 10%–20%, 5%–10%, respectively). The quantified values of the products are summarized in Table S2.

The primer-template substrates with or without the CTNAs used in the KF study, in addition to ddC, were treated with recombinant ERCC1-XPF in the same manner as described previously [24]. The results clearly indicated that the CTNA-containing oligonucleotides were cleaved by ERCC1-XPF (Figure 3A–F). The amounts of each product shown in lanes 3 in Figure 3A–F were quantified (Table S2), and the remarkable cleavage sites with the yield of >5% are indicated with triangles in each panel in Figure 3. Regardless of the CTNAs, the cleavage site 6 nt away from the terminal CTNAs, forming the 13-mer products (black triangles in Figure 3A–F), was common and the most prominent. The second major cleavage site was 7 nt away from the termini, forming 12-mer products, in most cases (Figure 3A–C,F). In the ABC- and CBV-containing substrates, however, it was 2 nt away from the termini, forming the 17-mer products (Figure 3D,E), even though the 12-mer products were also considerable. The minor cleavage sites producing the 9 and 18-mer products were diverse among the substrates (open triangles). These biochemical results demonstrate that ERCC1-XPF can remove the 3′-CTNAs by making incisions 2–7 nt away from the terminal modification.

**Figure 4:**
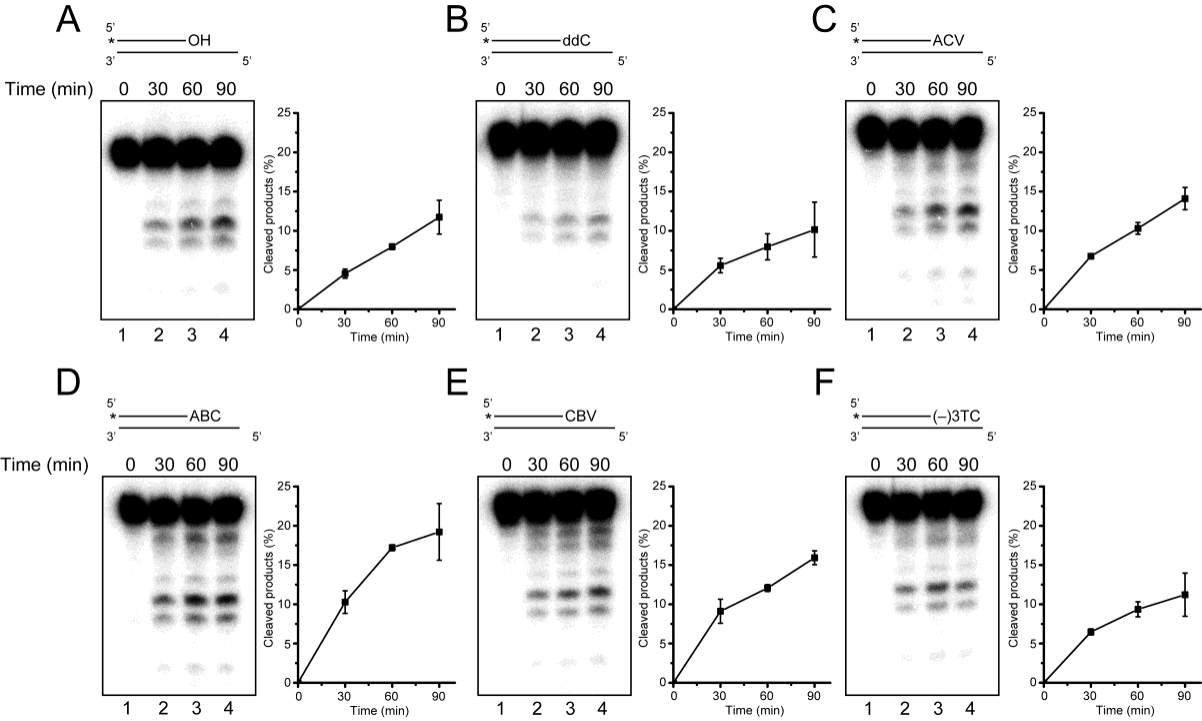
Time course of ERCC1-XPF cleavage. The ^32^P-labeled substrates (400 fmol) containing (**A**) 3′-OH; (**B**) ddC; (**C**) ACV; (**D**) ABC; (**E**) CBV; and (**F**) (−)3TC, were incubated at 30 °C for 0 (lanes 1), 30 (lanes 2), 60 (lanes 3) and 90 min (lanes 4), in the presence of ERCC1-XPF (92 fmol), in 10 µL of 50 mM Tris-HCl buffer (pH 8.0) containing 0.5 mM MnCl_2_, 0.5 mM DTT and 0.1 mg·mL^−1^ BSA. The experiments were performed in triplicate, and the increases in the amounts of the cleaved products shorter than the intact substrates were plotted. The data points are shown as mean ± SD.

To investigate the substrate specificity of ERCC1-XPF, time course experiments of the incisions were performed, and the cleaved products shorter than the intact substrates were plotted (Figure 4A–F). Consistent with the results in Figure 3, the 12- and 13-mer products were observed within 30 min in all of the tested substrates (lanes 2 in Figure 4). In the presence of ABC and CBV, the second major 17-mer product was also obtained within 30 min (Figure 4D,E). Overall, the cleaved products from the ABC- and CBV-containing substrates accumulated slightly faster than those from the unmodified oligonucleotide (Figure 4A). In contrast, the ddC modification was found to be the poorest substrate among the tested substrates, and its excision was slower than that of the unmodified substrate (Figure 4B). These differences indicated that ERCC1-XPF slightly prefers the abacavir-induced terminal modification.

### 2.5. ERCC1-XPF Associated Repair of CTNA-Induced DNA Damage

**Figure 5:**
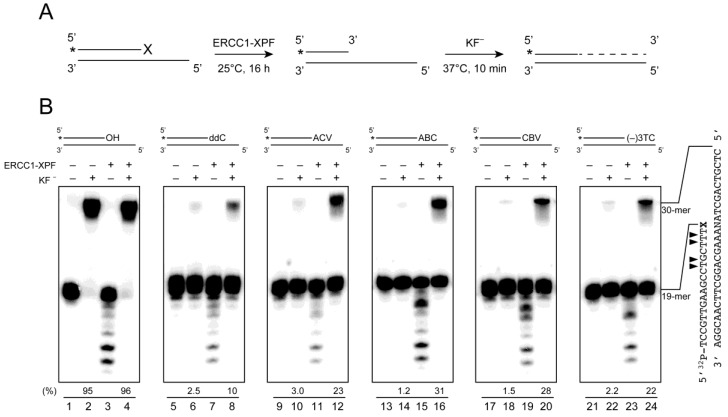
Repair of the CTNA-containing oligonucleotides by human ERCC1-XPF endonuclease and DNA polymerase. Panels A and B represent the reaction scheme and the results, respectively. First, the ^32^P-labeled substrates (400 fmol) were incubated at 25 °C for 16 h, in the absence (lanes 1, 2, 5, 6, 9, 10, 13, 14, 17, 18, 21 and 22) or presence (the other lanes) of ERCC1-XPF (230 fmol), in 10 µL of 50 mM Tris-HCl buffer (pH 8.0) containing 2 mM MgCl_2_, 0.5 mM DTT and 0.1 mg·mL^−1^ BSA. Then, a polymerase reaction mixture (5 µL, containing 30 mM Tris-HCl, (pH 7.9), 150 mM NaCl, 30 mM MgCl_2_, 30 mM DTT, 300 µM dNTPs and 0.1 unit of KF^−^ for even lanes) or 5 mM EDTA (5 µL for odd lanes) was added to the reaction mixture, and the total reaction mixtures (15 µL) were incubated at 37 °C for 10 min. The cleavage sites observed with ERCC1-XPF are indicated by black triangles. The fully extended products were quantified, and the values are shown.

As mentioned, 3′-CTNAs can be removed by TDP1 [10], which leaves a 3′-phosphate group at the 3′-terminus, whereas the incision of DNA by the ERCC1-XPF endonuclease restores a hydroxy group at this site [19], which enables the gap-filling DNA synthesis in the NER pathway. We assumed that DNA synthesis by DNA polymerases would resume after the excision of the 3′-CTNAs by ERCC1-XPF, leading to the completion of DNA repair. The ^32^P-labeled CTNA-containing substrates were first treated with ERCC1-XPF, and then with KF^−^ (Figure 5A). Note that Mg^2+^, instead of Mn^2+^, was used as a bivalent metal in this assay for the ERCC1-XPF cleavage activity. Although the highest activity was reportedly observed with Mn^2+^ [20], we employed Mg^2+^ in this particular case, since the presence of Mn^2+^ may modulate the processivity of DNA polymerases. Indeed, even in the presence of Mg^2+^, ERCC1-XPF cleaved the similar positions to those obtained with Mn^2+^ (lanes 3, 7, 11, 15, 19 and 23 in Figure 5B). In the successive treatment with ERCC1-XPF and KF^−^, the cleaved products shorter than the original oligonucleotides disappeared, and the fully-extended 30-mer products were observed (lanes 4, 8, 12, 16, 20 and 24). These results indicated that KF^−^ could resume DNA synthesis from the cleavage termini and extend the cleaved products by using the complementary strands as templates. The formation of the 30-mer products also suggested that the template strands were left intact and the CTNA-containing primers were selectively cleaved by ERCC1-XPF. The yields of the 30-mer products were quantified as 10% for ddC, 23% for ACV, 31% for ABC, 28% for CBV and 22% for (−)3TC (Figure 5B), which were in good agreement with the excision preferences shown in Figure 4B–F. In the absence of ERCC1-XPF, however, slight amounts (1%–3%) of the fully-extended 30-mer were observed after the treatment of CTNA-oligonucleotides with KF^−^ (lanes 6, 10, 14, 18 and 22), although KF^−^ could not resume DNA synthesis from the 3′-CTNA termini, as shown in Figure 2. This may be caused by the partial degradation of the oligonucleotides during the long incubation period (16 h before the addition of KF^−^), which would expose the 3′-hydroxy group by chance. Nonetheless, it is clear that the excision of the CTNAs by ERCC1-XPF facilitated the resumption of DNA synthesis by DNA polymerases.

## 3. Discussion

In this study, we synthesized four types of CTNA phosphoramidites (Scheme 1), and oligonucleotides containing CTNAs were successfully synthesized on the solid support. Our *in vitro* biochemical analysis revealed that these CTNAs were not efficiently removed by the exonuclease activity of the Klenow fragment (Figure 2), and that ERCC1-XPF could excise the 3′-CTNA (Figure 3 and Figure 4), reproducing the extendable 3′-OH terminus (Figure 5). 

In the TDP1-dependent repair pathway of the CTNA-induced DNA damage, the 3′-phosphate group, which blocks chain elongation by DNA polymerase, remains at the 3′-terminus after the removal in the TDP1-dependent pathway [10], and actually the repair cannot be completed only with TDP1. To complete the repair process, the 3′-phosphate should be processed by phosphatases, such as apurinic/apyrimidinic endonuclease 1 [25], polynucleotide kinase 3′-phosphatase [26], and aprataxin [27]. TDP1 displayed its diverse substrate specificities, and an *in vitro* analysis indicated that the exicision of 3′-AZT by TDP1 was ~100-fold slower than that of 3′-ACV [10], which was comparable to that of the normal 3′-T. This suggests the requirement of a backup mechanism that covers a wide range of CTNA-induced DNA damage.

In contrast to the TDP1-dependent repair, the cleavage of the CTNA strand by ERCC1-XPF directly produced the terminal 3′-hydroxy group, and the resumption of DNA replication by DNA polymerase was observed (Figure 5). These observations suggested that ERCC1-XPF dependent repair can be considered as a possible pathway for the CTNA-induced DNA damage. The time course of the strand cleavage by ERCC1-XPF indicated that the highest cleavage efficiency among the tested substrates was observed for the ABC-containing substrate, followed by the CBV-containing one (Figure 4), suggesting that terminal modifications bearing the carbohydrate backbone are recognized efficiently by ERCC1-XPF. The removal efficiencies of ddC, ACV, and (−)3TC were similar to those of the non-modified substrate. The wide substrate specificity of ERCC1-XPF may be beneficial for the repair of 3′-CTNAs that are left unrepaired in the TDP1-dependent process.

Among the CTNAs used in this study, ABC reportedly caused pronounced chromosomal breaks and induced the prolonged γH2AX activation associated with the co-localization of RAD51 in TDP1-deficient cells [11], suggesting that the double-strand break repair is induced in response to the ABC treatment. The homologous and non-homologous recombination processes reportedly involve 3′-end processing by the ERCC1-XPF endonuclease [28,29], and therefore it is possible that ERCC1-XPF functions in the cleavage of the 3′-CTNA strand via the recombination process, as an alternative pathway.

## 4. Materials and Methods

### 4.1. General Information

Acycloguanosine (acyclovir, ACV), [(1*S*,4*R*)-4-(2-amino-6-(cyclopropylamino)-9*H*-purin-9-yl)cyclopent-2-en-1-yl]methanol (abacavir, ABC) hemisulfate and (−)-β-l-2′,3′-dideoxy-3′-thia-cytidine (lamivudine, (−)3TC), were purchased from Sigma-Aldrich Co. (St. Louis, MO, USA), Carbosynth Ltd. (Berkshire, UK) and Wako Pure Chemical Industries, Ltd. (Osaka, Japan), respectively. Carbovir (CBV) was synthesized from optically pure (1*R*)-(−)-2-azabicyclo[2.2.1]hept-5-en-3-one (>95% e.e., Kanto Chemical Co., Inc., Tokyo, Japan), according to the previous reports [13,14]. Reagents for DNA synthesis were purchased from Glen Research (Sterling, VA, USA), and the other reagents and solvents were from Wako. TLC analyses were performed on Merck Silica gel 60 F_254_ TLC glass plates (Merck KGaA, Darmstadt, Germany), and the results were visualized by UV illumination at 254 nm. NMR and mass spectra were measured with INOVA500 (Agilent Technology, Santa Clara, CA, USA) and JMS-700 (JEOL Ltd., Tokyo, Japan) spectrometers, respectively. Chemical shifts for ^1^H- and ^31^P-NMR were calibrated with internal TMS and external trimethylphosphate, respectively. HPLC analyses were performed with a Gilson gradient-type analytical system (Gilson, Inc., Middleton, WI, USA) equipped with a Waters 2996 photodiode-array detector, and a µBondasphere C18 column (Waters Co., Milford, MA, USA) was used on this system at a flow rate of 1.0 mL·min^−1^, with a linear gradient of acetonitrile in 0.1 M triethylammonium acetate generated over 20 min.

### 4.2. Synthesis of CTNA Phosphoramidites

#### 4.2.1. Synthesis of 5′-*O*-TBS-abacavir (**5**)

Abacavir hemisulfate (332 mg, 990 µmol) and imidazole (167 mg, 2.45 mmol) were coevaporated with anhydrous pyridine three times, and then suspended in anhydrous pyridine (8 mL). To the mixture, *tert*-butyldimethylsilyl chloride (190 mg, 1.26 mmol) was added. The reaction mixture was diluted with ethyl acetate (100 mL) after continuous stirring for 20 h at room temperature, and the organic layer was washed with saturated NaHCO_3_ (80 mL × 2) and brine (80 mL × 1), and then was dried over Na_2_SO_4_. The solvent was evaporated *in vacuo*. The residue was co-evaporated with toluene three times, and the product was purified with silica gel column chromatography with a stepwise gradient of 0%–2% methanol in chloroform. The fractions containing the product were evaporated to give a white foamy solid. Yield 367 mg (916 µmol, 93%). TLC (CHCl_3_/MeOH, 10:1) *R*_f_ 0.54. ^1^H-NMR (500 MHz, CDCl_3_) δ (ppm) 7.54 (s, 1H), 6.11 (dt, *J* = 5.5 and 2 Hz, 1H), 5.83 (dt, *J* = 6 and 2.2 Hz, 1H), 5.63 (m, 1H), 5.55 (tt, *J* = 5 and 2.2 Hz, 1H), 4.71 (br, 2H), 3.68 (dd, *J* = 10 and 5.8 Hz, 1H), 3.60 (dd, *J* = 10 and 5.8 Hz, 1H), 2.99 (m, 2H), 2.74 (dt, *J* = 13.5 and 8.8 Hz, 1H), 0.89 (s, 9H), 0.86 (m, 2H), 0.61 (m, 2H), 0.05 (s, 6H). FAB-HRMS *m*/*z* 401.2487 ([M + H]^+^, *m*/*z* 401.2485 calcd for C_20_H_33_ON_6_Si) (See Figure S5).

#### 4.2.2. Synthesis of *N*^2^-Isobutyryl-5′-*O*-TBS-abacavir (**6**)

Compound **5** (344 mg, 858 µmol) was coevaporated with anhydrous pyridine three times, and then dissolved in anhydrous pyridine (5 mL). The solution was cooled in an ice bath, and then isobutyryl chloride (108 µL, 1.03 mmol) was added. The ice bath was removed, and the reaction mixture was stirred for 1 h at room temperature. The reaction mixture was diluted with ethyl acetate (100 mL), and the organic layer was washed with saturated NaHCO_3_ (100 mL × 2) and brine (100 mL × 1), and then was dried over Na_2_SO_4_. The solvent was evaporated *in vacuo*. The residue was co-evaporated with toluene three times, and the product was purified with silica gel column chromatography with a stepwise gradient of 20%–60% ethyl acetate in hexane. The fractions containing the product were evaporated to give a white solid. Yield 357 mg (759 µmol, 88%). TLC (hexane/ethyl acetate, 3:2) *R*_f_ 0.21. ^1^H-NMR (500 MHz, CDCl_3_) δ (ppm) 7.78 (s, 1H), 7.72 (s, 1H), 6.13 (dt, *J* = 5.5 and 2.2 Hz, 1H), 5.84 (m, 2H), 5.64 (m, 1H), 3.70 (dd, *J* = 10 and 5.5 Hz, 1H), 3.60 (dd, *J* = 10 and 5.5 Hz, 1H), 2.99 (m, 2H), 2.77 (dt, *J* = 13.5 and 8.8 Hz, 1H), 1.66 (m, 3H), 1.27 (s × 2, 3H × 2), 0.89 (s, 9H), 0.86 (m, 2H), 0.63 (m, 2H), 0.05 (s, 6H). FAB-HRMS *m*/*z* 471.2902 ([M + H]^+^, *m*/*z* 471.2904 calcd for C_24_H_39_O_2_N_6_Si) (See Figure S6).

#### 4.2.3. Synthesis of *N*^2^-Isobutyryl-abacavir (**7**)

Compound **6** (344 mg, 732 µmol) was dissolved in anhydrous THF (5 mL), and the solution was cooled in an ice bath. TEA·3HF (447 µL, 2.93 mmol) was added to the solution. The ice bath was removed, and the reaction mixture was stirred overnight at room temperature. The reaction mixture was diluted with chloroform (100 mL), and the organic layer was washed with saturated NaHCO_3_ (100 mL × 2) and brine (100 mL × 1), and then was dried over Na_2_SO_4_. The solvent was evaporated *in vacuo*. The residue was co-evaporated with toluene three times, and the product was purified with silica gel column chromatography with a stepwise gradient of 0%–4% methanol in chloroform. The fractions containing the product were evaporated to give a white solid. Yield 231 mg (648 µmol, 89%). The product was dissolved in 20% methanol in chloroform (2.5 mL), and the solution was dropped into the mixture of hexane and diethyl ether (50 mL, 1/1, *v*/*v*) to give a white powdery product. TLC (CHCl_3_/MeOH, 10:1) *R*_f_ 0.29. ^1^H-NMR (500 MHz, CDCl_3_) δ (ppm) 7.79 (s, 1H), 7.71 (s, 1H), 6.14 (dt, *J* = 5.5 and 2.2 Hz, 1H), 5.91 (br, 1H), 5.86 (dt, *J* = 5.5 and 2.2 Hz, 1H), 5.58 (m, 1H), 3.84 (m, 1H), 3.72 (m, 1H), 3.09 (m, 1H), 3.03 (br, 1H), 2.80 (dt, *J* = 14 and 9.2 Hz, 1H), 2.01 (dt, *J* = 14 and 6 Hz, 1H), 1.27 (s, 3H), 1.26 (s, 3H), 0.87 (m, 2H), 0.63 (m, 2H). FAB-HRMS *m*/*z* 357.2037 ([M + H]^+^, *m*/*z* 357.2039 calcd for C_18_H_25_O_2_N_6_) (See Figure S7).

#### 4.2.4. Synthesis of *N*^2^-Isobutyryl-abacavir phosphoramidite (**1**)

Compound **7** (37 mg, 104 µmol) was suspended in anhydrous THF (1.0 mL), and subsequently diisopropylethylamine (72 µL, 416 µmol) and 2-cyanoethyldiisopropylchlorophosphoramidite (47 µL, 208 µmol) were added. The reaction mixture turned into a clear solution, and then precipitates appeared. After continuous stirring for 30 min at room temperature, the reaction mixture was diluted with ethyl acetate (40 mL). The organic layer was washed with 2% NaHCO_3_ (40 mL × 2) and brine (40 mL × 1), and then was dried over Na_2_SO_4_. The solvent was evaporated *in vacuo*. The residue was co-evaporated with toluene once, and the product was purified with silica gel column chromatography with a stepwise gradient of 50%–70% ethyl acetate in hexane containing 0.1% pyridine. The fractions containing the product were evaporated to dryness. The residue was further coevaporated with acetonitrile to give a glassy product. Yield 38 mg (58 µmol, 56%, estimated by ^31^P-NMR). TLC (CHCl_3_/MeOH, 10:1) *R*_f_ 0.78. ^31^P-NMR (203 MHz, acetone-*d*_6_) δ (ppm) 145.49 and 145.37. FAB-HRMS *m*/*z* 557.3115 ([M + H]^+^, *m*/*z* 557.3117 calcd for C_27_H_42_O_3_N_8_P) (See Figure S8).

#### 4.2.5. Synthesis of *N*^2^-DMF-carbovir (**8**)

Optically pure carbovir was synthesized from (1*R*)-(−)-2-azabicyclo[2.2.1]hept-5-en-3-one according to the previous reports [13,14]. Carbovir (410 mg, 1.66 mmol) was coevaporated with anhydrous DMF three times, and the residue was dissolved in DMF (8 mL). To the solution, *N,N*-dimethylformamide dimethyl acetal (1.1 mL, 8.24 mmol) was added, and the resulting solution was stirred overnight at room temperature. The solvent was removed *in vacuo*, and the residue was co-evaporated with toluene three times, and the product was purified with silica gel column chromatography with a stepwise gradient of 0%–6% methanol in chloroform. The fractions containing the product were evaporated to dryness. Yield 475 mg (1.57 mmol, 95%). The product was dissolved in chloroform/methanol solution (4 mL, 10/1, *v*/*v*), and the solution was dropped into hexane/diethyl ether solution (80 mL, 1/1, *v*/*v*) to give a powdery product. TLC (CHCl_3_/MeOH, 10:1) *R*_f_ 0.29. ^1^H-NMR (500 MHz, DMSO-*d*_6_) δ (ppm) 11.2 (s, 1H), 8.59 (s. 1H), 7.72 (s, 1H), 6.11 (dt, *J* = 5.5 and 2 Hz, 1H), 5.86 (dt, *J* = 6 and 2.2 Hz, 1H), 5.51 (m, 1H), 4.71 (t, *J* = 5.5 Hz, 1H), 3.47 (td, *J* = 5.5 and 2 Hz, 2H), 3.15 (s, 3H), 3.03 (s, 3H), 2.88 (m, 1H), 2.65 (dt, *J* = 14 and 8.7 Hz, 1H), 1.64 (dt, *J* = 13.5 and 6.2 Hz, 1H). FAB-HRMS *m*/*z* 303.1573 ([M + H]^+^, *m*/*z* 303.1569 calcd for C_14_H_19_O_2_N_6_) (See Figure S9).

#### 4.2.6. Synthesis of *N*^2^-DMF-Carbovir phosphoramidite (**2**)

Compound **8** (62 mg, 206 µmol) was suspended in anhydrous acetonitrile (1.5 mL), and subsequently diisopropylethylamine (144 µL, 824 µmol) and 2-cyanoethyldiisopropylchloro-phosphoramidite (92 µL, 206 µmol) were added. After continuous stirring for 30 min at room temperature, the reaction mixture was diluted with ethyl acetate (40 mL). The organic layer was washed with 2% NaHCO_3_ (40 mL × 2) and brine (40 mL × 1), and then was dried over Na_2_SO_4_. The solvent was evaporated *in vacuo*. The residue was co-evaporated with toluene once, and the product was purified with silica gel column chromatography with a stepwise gradient of 0%–3% methanol in chloroform containing 0.5% pyridine. The fractions containing the product were evaporated to dryness. The residue was further co-evaporated with acetonitrile to give an oily product. Yield 71 mg (115 µmol, 56%, estimated by ^31^P-NMR). TLC (CHCl_3_/MeOH, 10:1) *R*_f_ 0.62. ^31^P-NMR (203 MHz, acetone-*d*_6_) δ (ppm) 145.48 and 145.33. FAB-HRMS *m*/*z* 503.2650 ([M + H]^+^, *m*/*z* 503.2648 calcd for C_23_H_36_O_3_N_8_P) (See Figure S10).

#### 4.2.7. Synthesis of *N*^2^-DMF-Acyclovir phosphoramidite (**3**)

*N*^2^-dmf-acyclovir **9** was synthesized according to the previous report [16]. *N*^2^-dmf-acyclovir **9** (56 mg, 200 µmol) was coevaporated with anhydrous THF three times, and suspended in a mixture of anhydrous THF and DMF (1.8 mL, 2/1, *v*/*v*). To the suspension, diisopropylethylamine (139 µL, 800 µmol) and 2-cyanoethyldiisopropylchlorophosphoramidite (89 µL, 400 µmol) were added. After continuous stirring for 30 min at room temperature, the reaction mixture turned into a clear solution, and the mixture was diluted with ethyl acetate (40 mL). The organic layer was washed with 2% NaHCO_3_ (40 mL × 2) and brine (40 mL × 1), and then was dried over Na_2_SO_4_. The solvent was evaporated *in vacuo*. The residue was co-evaporated with toluene once, and the product was purified with silica gel column chromatography with a stepwise gradient of 0%–4% methanol in chloroform containing 0.5% pyridine. The fractions containing the product were evaporated to dryness. The residue was further coevaporated with acetonitrile to give a glassy product. Yield 24 mg (49 µmol, 25%). TLC (CHCl_3_/MeOH, 10:1) *R*_f_ 0.30. ^31^P-NMR (203 MHz, acetone-*d*_6_) δ 145.97 ppm. FAB-HRMS *m*/*z* 481.2432 ([M + H]^+^, *m*/*z* 481.2441 calcd for C_20_H_34_O_4_N_8_P) (See Figure S11).

#### 4.2.8. Synthesis of (−)-β-l-*N*^4^-Benzoyl-2’,3′-dideoxy-3′-thiacytidine (**10**)

Lamivudine (96 mg, 419 µmol) and benzoic anhydride (187 mg, 830 µmol) were suspended in anhydrous ethanol (2.0 mL) in a glass vial, and the vial was tightly sealed with a screw cap. The reaction mixture was heated at 70 °C for 2 h, and then benzoic anhydride (95 mg, 420 µmol) was added to the solution to complete the reaction. After 30 min, the reaction mixture was diluted with chloroform (40 mL). The organic layer was washed with saturated NaHCO_3_ (40 mL × 2) and brine (40 mL × 1), and then was dried over Na_2_SO_4_. The solvent was concentrated *in vacuo*. The residue was triturated with diethyl ether, and the precipitate was collected and washed with diethyl ether to give a white powdery product. Yield 112 mg (335 µmol, 80%). TLC (CHCl_3_/MeOH, 10:1) *R*_f_ 0.50. ^1^H-NMR (500 MHz, DMSO-*d*_6_) δ (ppm) 11.24 (br, 1H), 8.45 (d, *J* = 7.5 Hz, 1H), 8.01 (m, 2H), 7.63 (m, 1H), 7.52 (m, 2H), 7.35 (m, 1H), 6.25 (dd, *J* = 5.8, 3.3 Hz, 1H), 5.43 (t, *J* = 5.8 Hz, 1H), 5.28 (t, *J* = 4.3 Hz, 1H), 3.85 (m, 2H), 3.59 (dd, *J* = 12.5, 5.5 Hz, 1H), 3.25 (m, 1H). FAB-HRMS *m*/*z* 334.0860 ([M + H]^+^, *m*/*z* 334.0862 calcd for C_15_H_16_O_4_N_3_S) (See Figure S12).

#### 4.2.9. Synthesis of *N*^4^-Benzoyl-lamivudine phosphoramidite (**4**)

Compound **10** (36 mg, 107 µmol) was co-evaporated with anhydrous THF three times, and suspended in anhydrous THF (1.0 mL). To the suspension, diisopropylethylamine (75 µL, 428 µmol) and 2-cyanoethyldiisopropylchlorophosphoramidite (48 µL, 214 µmol) were added. The reaction mixture was stirred for 30 min at room temperature, and then additional diisopropylethylamine (37 µL, 212 µmol) and 2-cyanoethyldiisopropylchlorophosphoramidite (24 µL, 108 µmol) were added to complete the reaction. After 10 min, the reaction mixture was diluted with ethyl acetate (40 mL). The organic layer was washed with 2% NaHCO_3_ (40 mL × 2) and brine (40 mL × 1), and then was dried over Na_2_SO_4_. The solvent was evaporated *in vacuo*. The residue was co-evaporated with toluene once, and the product was purified with silica gel column chromatography with a stepwise gradient of 40%–80% ethyl acetate in hexane containing 0.1% pyridine. The fractions containing the product were evaporated to dryness. The residue was further coevaporated with acetonitrile to give a glassy product. Yield 54 mg (76 µmol, 71%, estimated by ^31^P-NMR). TLC (CHCl_3_/MeOH, 10:1) *R*_f_ 0.80. ^31^P-NMR (203 MHz, acetone-*d*_6_) δ (ppm) 147.12, 147.03. FAB-HRMS *m*/*z* 534.1938 ([M + H]^+^, *m*/*z* 534.1940 calcd for C_24_H_33_O_5_N_5_PS) (See Figure S13).

### 4.3. Synthesis of Oligonucleotides Containing CTNAs at the 3′-Termini

The phosphoramidite reagents of the CTNAs **1**–**4** were dissolved in anhydrous acetonitrile at a concentration of 0.11 M, and were installed on a 3400 DNA synthesizer (Applied Biosystems, Waltham, MA, USA). Solutions of nucleoside phosphoramidites for the 5′ → 3′ synthesis and 2′,3′-dideoxycytidine (ddC) phosphoramidite (Glen Research) were also installed. An 18-mer oligonucleotide, d(TCCGTTGAAGCCTGCTTT), was first synthesized in the 5′ → 3′ direction on a 0.2 µmol scale. After the removal of the last 3′-DMT group, the phosphoramidite building blocks of the CTNAs were allowed to react with the exposed 3′-hydroxy group in the presence of tetrazole, on the DNA synthesizer. This process was repeated twice, while omitting the detritylation, capping, and oxidation steps. Subsequently, the supports were treated with an iodine solution to oxidize the newly-generated phosphite triester into the phosphotriester group, to yield the CTNA-attached 19-mer oligonucleotides, d(TCCGTTGAAGCCTGCTTT)X, where X represents the CTNAs and ddC. The synthesized oligonucleotides were treated with 28% ammonia water for 1 h at room temperature. The ammoniac solutions were transferred into glass vials with screw caps, and were heated at 55 °C for 5 h (CBV, (−)3TC and ACV) or 24 h (ABC). After evaporation of the solution, the desired oligonucleotides containing the CTNAs were purified by HPLC, with linear gradients of 5%–13% or 6%–14% acetonitrile. The oligonucleotides were characterized by LC-ESI mass spectrometry (Table S1 and Figures S1–S4).

To quantify the purified oligonucleotides, aliquots of the CTNA-oligonucleotide solutions, in addition to the quantified solution of the oligonucleotide without the CTNA modification, were dried with a SpeedVac centrifugal evaporator, and the residues were treated with S1 nuclease (180 units, Takara Bio, Inc.) in 20 µL of 30 mM sodium acetate buffer (pH 4.6) containing 100 mM NaCl and 1 mM ZnCl_2_ at 37 °C. After 24 h, the reaction was quenched by addition of 5 µL of 500 mM Tris-HCl buffer (pH 9.0) containing 10 mM MgCl_2_, and 2 µL of alkaline phosphatase from *Escherichia coli* C75 (1.0 unit, Takara Bio) and 23 µL of water were added. The total mixtures (50 µL) were incubated at 37 °C for 2 h. The obtained nucleosides were analyzed by HPLC, and an ODS-3 C18 column (GL Science Inc., Tokyo, Japan) was used at a flow rate of 1.0 mL·min^−1^ at ambient temperature, with a linear gradient of 2.5%–15% acetonitrile in 0.1 M triethylammonium acetate generated over 20 min. By comparing the peak areas of dC and dG detected at 260 nm, concentrations of the CTNA-containing oligonucleotides were calculated.

### 4.4. General Procedure for the Biochemical Experiments

The 5′-^32^P-labeled oligonucleotides were prepared by treating the CTNA-oligonucleotides (100 nmol) with T4 polynucleotide kinase (10 units, Takara Bio) at 37 °C for 30 min, in 70 mM Tris-HCl buffer (pH 7.6) containing 10 mM MgCl_2_, 5 mM DTT and γ-^32^P adenosine triphosphate (~400 kBq, Perkin Elmer, Waltham, MA, USA). The reaction mixture was heated at 95 °C for 5 min, and was then passed through a MicroSpin^TM^ G-25 column (GE Healthcare, Buckinghamshire, UK). Aliquots (20 pmol) of the obtained ^32^P-labeled oligonucleotides were mixed in water with the complementary strands (20 pmol), d(CTCGTCAGCTANAAAGCAGGCTTCAACGGA), where N represents A, G or C, depending on the type of CTNA. The solutions were heated at 80 °C for 2 min, and then were gradually cooled down to 4 °C over 2 h. The hybridized oligonucleotides were used for the enzymatic reaction. After the reaction, an equivalent amount of stop solution, containing 95% formamide, 20 mM EDTA, 0.025% bromophenol blue and 0.025% xylene cyanol, was added to the reaction mixture. The mixtures were heated at 95 °C for 10 min, and then immediately cooled in ice. The reaction products were separated by electrophoresis on a 20% polyacrylamide/7.5 M urea gel. The dried gels were visualized on a GE Healthcare FLA7000 image analyzer. The band intensities in the obtained gel images were quantified by the MultiGauge ver. 3.0 software (Fuji Film, Tokyo, Japan).

### 4.5. Removal of the CTNAs by ERCC1-XPF Endonucleases

The recombinant human ERCC1-XPF endonuclease was prepared according to the previous report [24]. The concentration of the ERCC1-XPF solution was determined by the Bradford method, with Quick Start^TM^ Bradford 1 × Dye Reagent and Quick Start^TM^ Bovine Serum Albumin Standard (Bio-Rad Laboratories, Hercules, CA, USA). The hybridized oligonucleotides (400 fmol) were treated with ERCC1-XPF in 10 µL of 50 mM Tris-HCl buffer (pH 8.0), containing 0.5 mM MnCl_2_, 0.5 mM DTT and 0.1 mg·mL^−1^ BSA, at 30 °C for 90 min. The products were analyzed by denaturing PAGE. Time course experiments were performed in the same reaction mixture, except for the enzyme amount (92 fmol), by varying the incubation time (0, 30, 60, 90 min).

### 4.6. Resumption of DNA Synthesis by DNA Polymerase after the Removal of CTNAs

The hybridized oligonucleotides (400 fmol) were first treated with ERCC1-XPF (230 fmol) in 10 µL of 50 mM Tris-HCl buffer (pH 8.0), containing 2 mM MgCl_2_, 0.5 mM DTT and 0.1 mg·mL^−1^ BSA, at 25 °C for 16 h. To the reaction mixture was added 5 µL of 30 mM Tris-HCl buffer (pH 7.9), containing 150 mM NaCl, 30 mM MgCl_2_, 30 mM DTT, 300 µM dNTPs and the Klenow fragment of *Escherichia coli* DNA polymerase I, lacking the 3′–5′ exonuclease activity (KF^−^, 0.1 unit, Takara Bio). The total reaction mixtures (15 µL) were incubated at 37 °C for 10 min, and the products were analyzed by denaturing PAGE.

## 5. Conclusions

We successfully synthesized the 3′-CTNA oligonucleotides on a solid support, and used them to investigate the repair of the CTNA-induced DNA damage by ERCC1-XPF endonuclease. The results indicated that ERCC1-XPF can remove the 3′-CTNAs, to expose the re-extendable 3′-OH. Since CTNAs would be metabolized in various ways, depending on their specific characteristics, the repair pathways of the CTNA-induced DNA damage would also be diverse, and our results suggested that structure-specific endonuclease-dependent repair is a possible pathway for the repair of the CTNA-induced DNA damage. Although it is still unclear whether ERCC1-XPF is involved in the repair *in vivo*, cell biological studies will address the involvement of ERCC1-XPF in the repair of the CTNA-induced damage in the future.

## Supplementary Materials

Supplementary materials can be accessed at: http://www.mdpi.com/1420-3049/21/6/766/s1.

## Abbreviations

The following abbreviations are used in this manuscript:

3TC	2’,3′-dideoxy-3′-thiacytidine
ABC	Abacavir
ACV	Acyclovir
AZT	3′-deoxy-3′-azidothymidine
BSA	Bovine Serum Albumin
bz	Benzoyl
CBV	Carbovir
CTNAs	Chain-terminating Nucleoside Analogs
ddC	2′,3′-dideoxycytidine
DIPEA	Diisopropylethylamine
dmf	*N,N*-dimethylformamidyl
dNTPs	2’-deoxynucleoside triphosphates
DTT	Dithiothreitol
ERCC1-XPF	Excision repair cross complementing protein 1–xeroderma pigmentosum group F
FAB-HRMS	Fast atom bombardment-High resolution mass spectroscopy
ibu	Isobutyryl
KF	Klenow fragments of *Escherichia coli* DNA polymerase I
mtDNA	Mitochondrial DNA
NER	Nucleoside excision repair
nt	Nucleotide
PAGE	Polyacrylamide gel electrophoresis
phos	2-cyanoethyl-*N,N*-diisopropylphosphoramidyl
Polγ	DNA polymerase γ
RT	Reverse transcriptase
SD	Standard deviation
TBS	*tert*-butyldimethylsilyl
TDP1	Tyrosyl-DNA phosphodiesterase 1
TEA	Triethylamine
