# Supplementary material for: Chemical Incorporation of Chain-Terminating Nucleoside Analogs as 3′-Blocking DNA Damage and Their Removal by Human ERCC1-XPF Endonuclease
Source: Molecules. 2016 Jun 11;21(6):766. doi: 10.3390/molecules21060766 (PMC6273010; doi:10.3390/molecules21060766)
Supplement: Supplementary file 1 [file molecules-21-00766-s001.pdf]

Supplementary Materials: Chemical Incorporation of Chain-Terminating Nucleoside Analogs as 3′-Blocking DNA Damage and Their Removal by Human ERCC1-XPF Endonuclease

Junpei Yamamoto, Chiaki Takahata, Isao Kuraoka, Kouji Hirota and Shigenori Iwai

Table S1. ESI-MS analysis of the synthesized oligonucleotides

Entry	Sequence (5′→3′)	Observed *m*/*z*	Calculated *m*/*z*[a]	Yield[b]
ACV 19-mer	TCC GTT GAA GCC TGC TTT ACV	5743.51	5742.74	62%
ABC 19-mer	TCC GTT GAA GCC TGC TTT ABC	5804.79	5803.87	45%
CBV 19-mer	TCC GTT GAA GCC TGC TTT CBV	5765.50	5765.79	32%
(–)3TC 19-mer	TCC GTT GAA GCC TGC TTT (–)3TC	5747.65	5746.78	70%

[a]Mass unit was calculated as $[M–H]^-$ form.

[b]Isolated yields.

Table S2. Cleavage of the primer-template substrates by ERCC1-XPF

Products	-OH (%)[a]	ddC (%)[a]	ACV (%)[a]	ABC (%)[a]	CBV (%)[a]	(–)3TC (%)[a]
19-mer	–	36.3[b]	32.8[b]	11.3[b]	12.4[b]	42.9[b]
18-mer	27.6[b]	5.2	5.2	2.3	2.7	5.4
17-mer	2.3	7.5	3.2	17.7	15.3	5.4
16-mer	3.5	7.5	9.8	5.8	11.7	12.4
15-mer	1.3	1.6	1.7	1.6	2.4	1.7
14-mer	4.8	3.1	3.8	4.7	4.5	2.9
13-mer	28.6	19.7	22.9	28.7	25.0	17.1
12-mer	16.7	11.8	11.3	15.3	13.8	8.1
11-mer	1.9	1.6	1.4	1.7	2.0	1.0
10-mer	1.9	0.8	1.1	1.5	1.4	0.4
9-mer	8.8	3.6	5.2	7.2	6.4	2.0
8-mer	1.7	0.8	1.0	1.3	1.5	0.4
7-mer	1.0	0.5	0.6	0.9	0.9	0.3

[a]The band intensities of each of the products in lanes 3 in Figure 3A–F were quantified.

[b]The original primers.

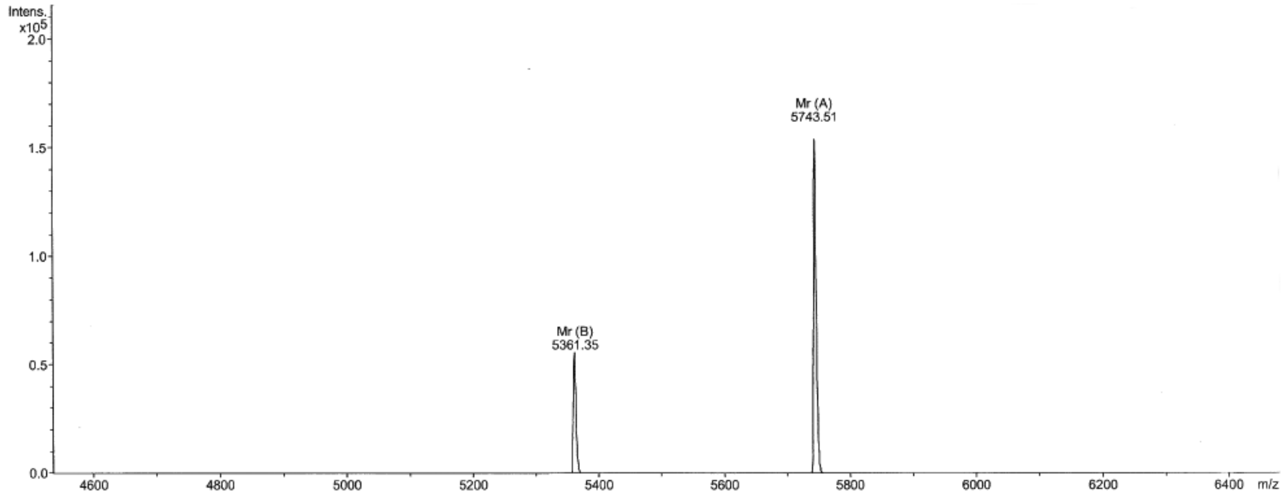

Figure S1. ESI mass spectrum of ACV 19-mer with the internal standard (calcd. *m*/*z* 5361.56).

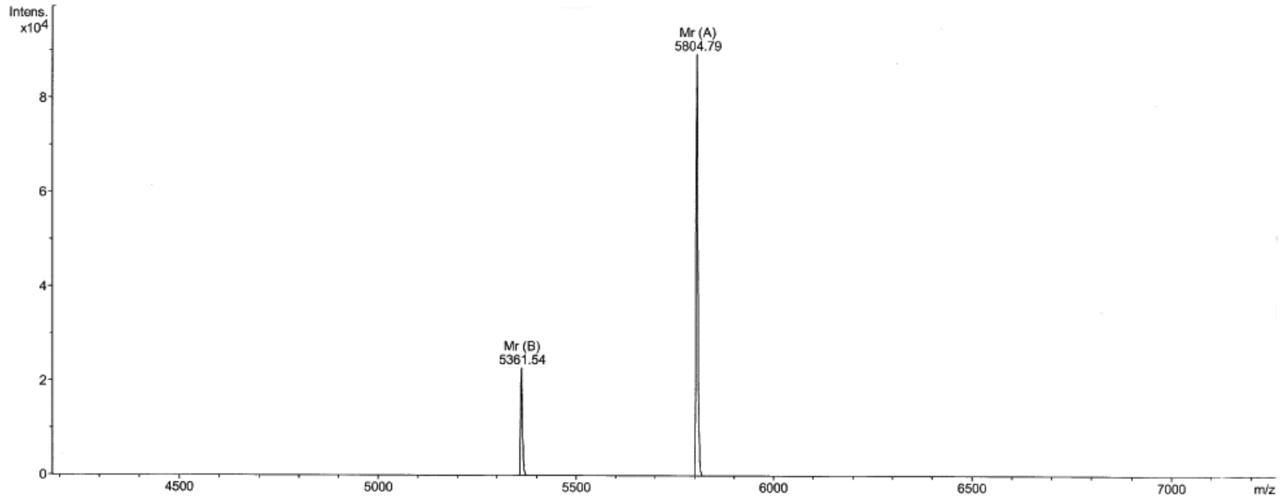

Figure S2. ESI mass spectrum of ABC 19-mer with the internal standard (calcd. *m*/*z* 5361.56).

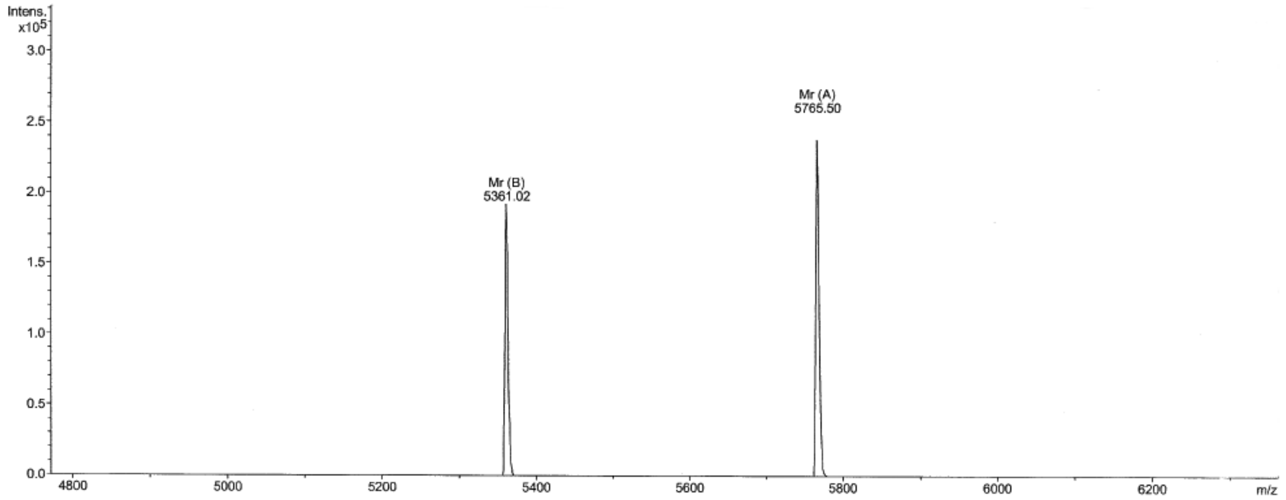

Figure S3. ESI mass spectrum of CBV 19-mer with the internal standard (calcd. *m*/*z* 5361.56).

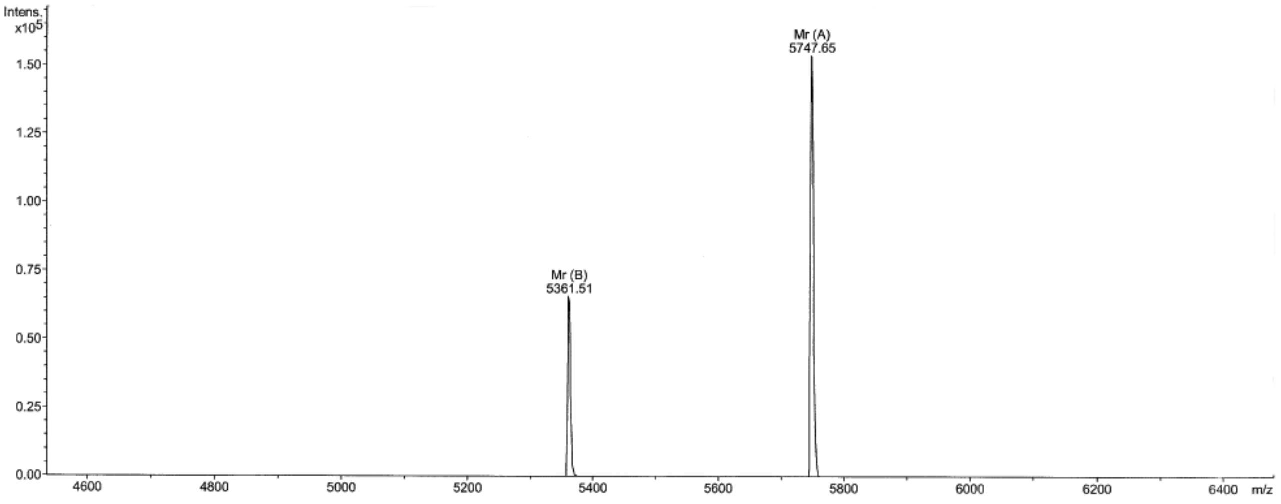

Figure S4. ESI mass spectrum of (–)3TC 19-mer with the internal standard (calcd. *m*/*z* 5361.56).

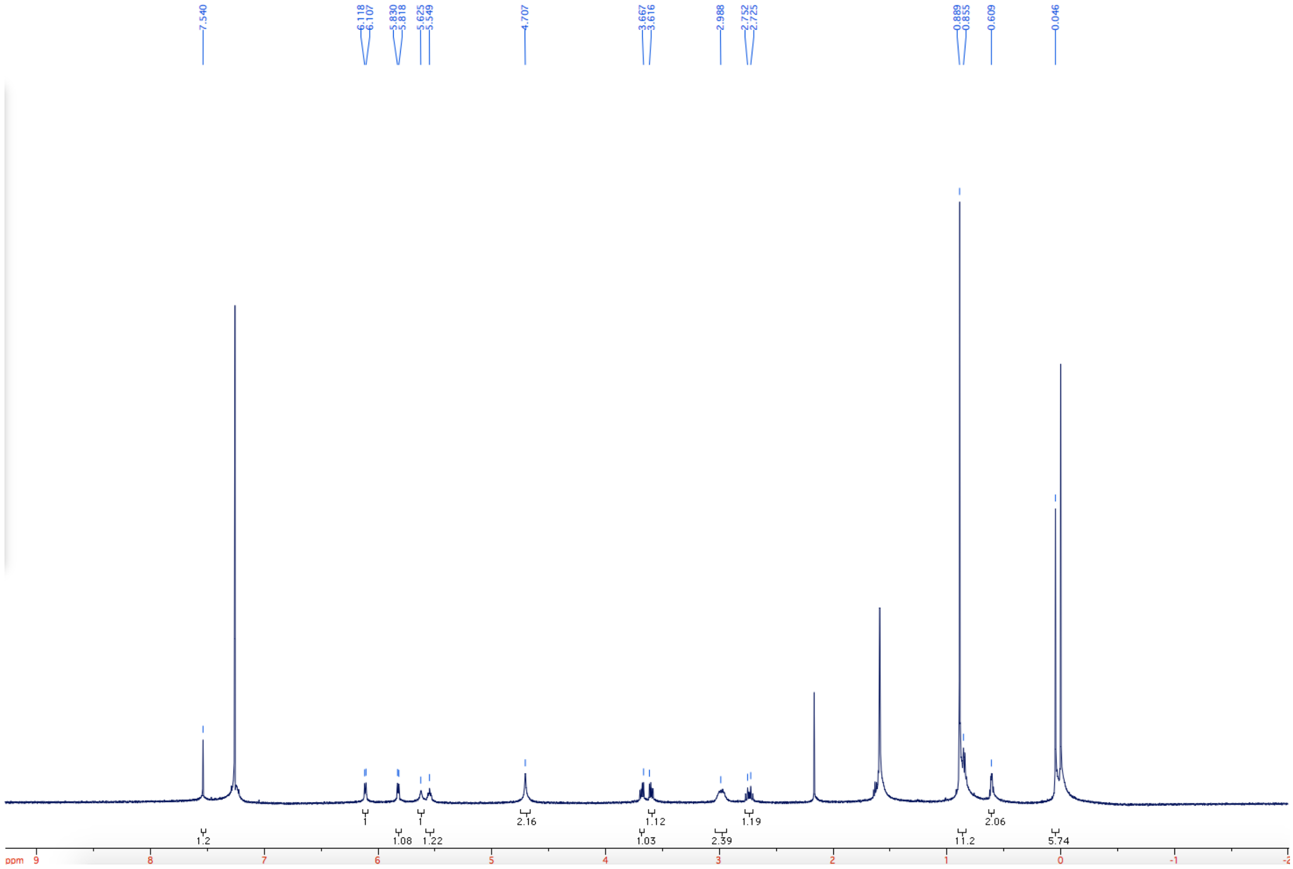

Figure S5. ^{1}H NMR spectrum of compound **5**.

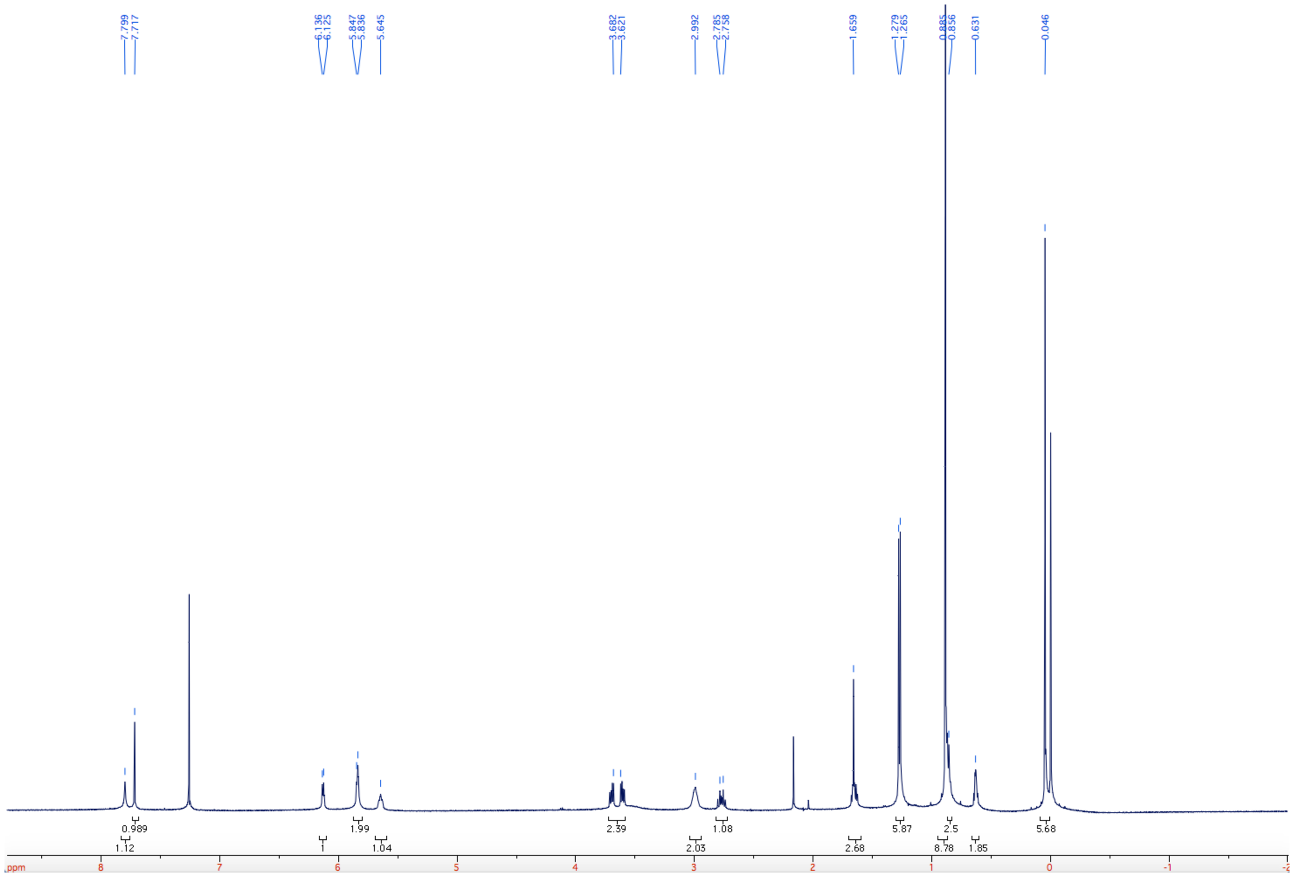

Figure S6. ^{1}H NMR spectrum of compound **6**.

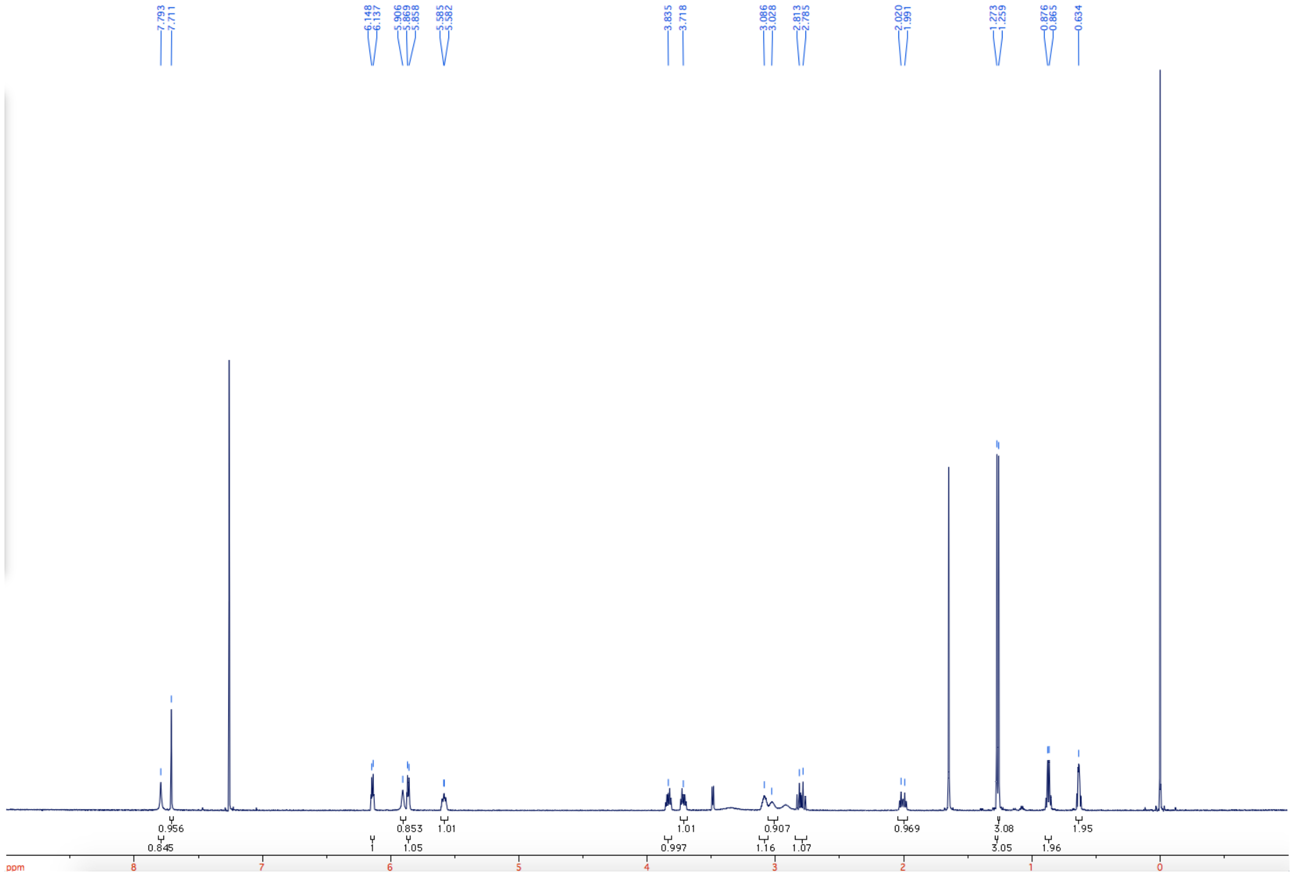

Figure S7. ^{1}H NMR spectrum of compound **7**.

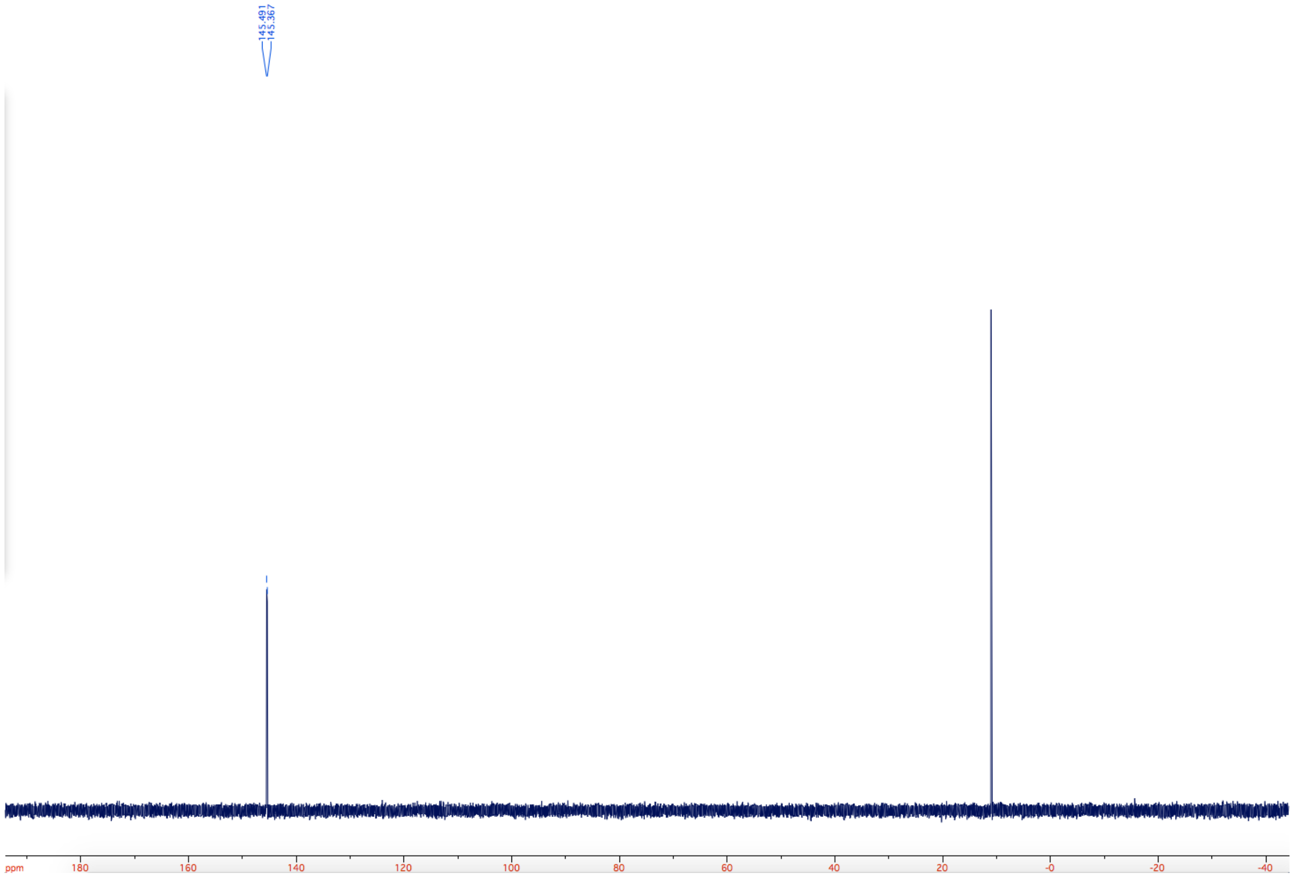

Figure S8. ^{31}P NMR spectrum of compound **1**. The signal at 11 ppm comes from an impurity.

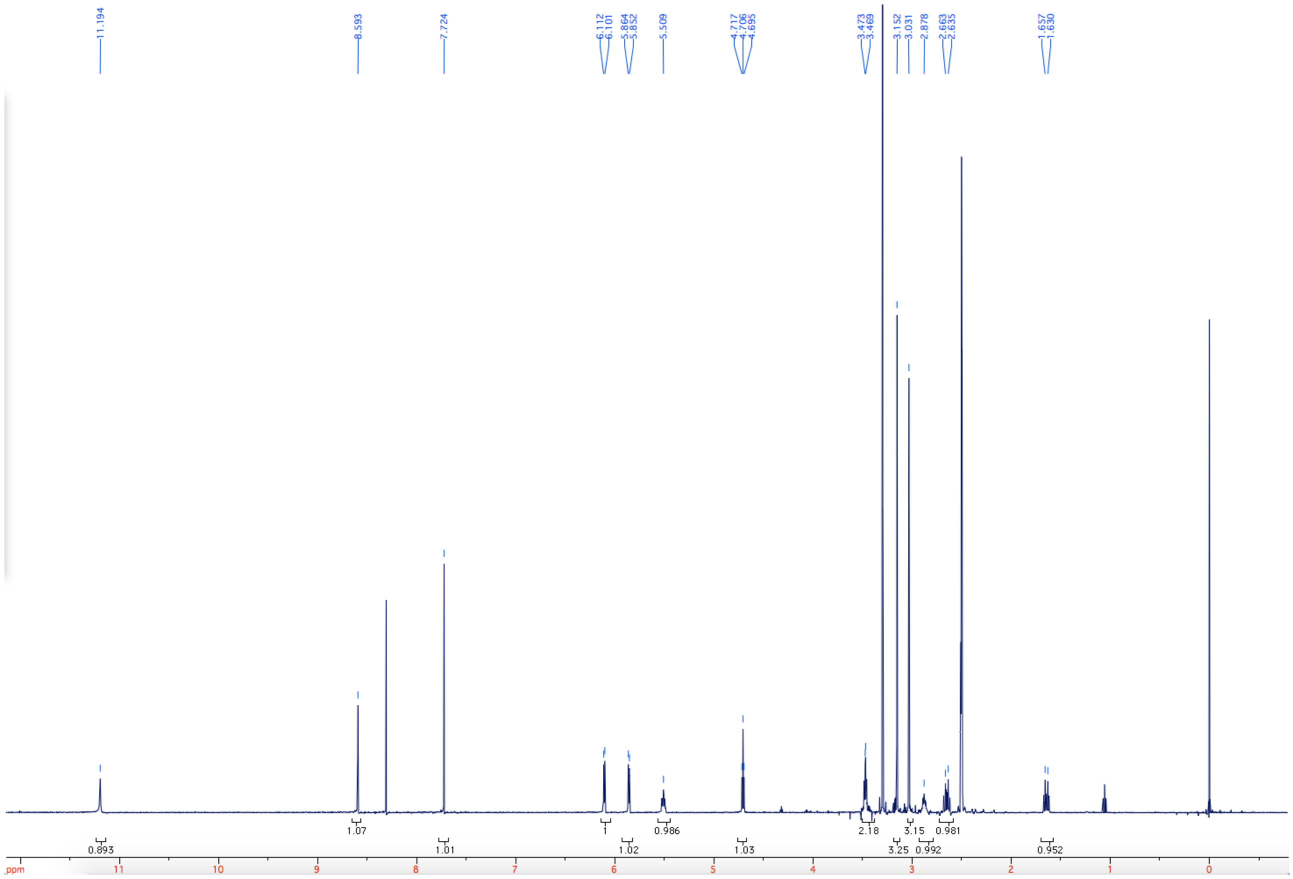

Figure S9. ^{1}H NMR spectrum of compound **8**.

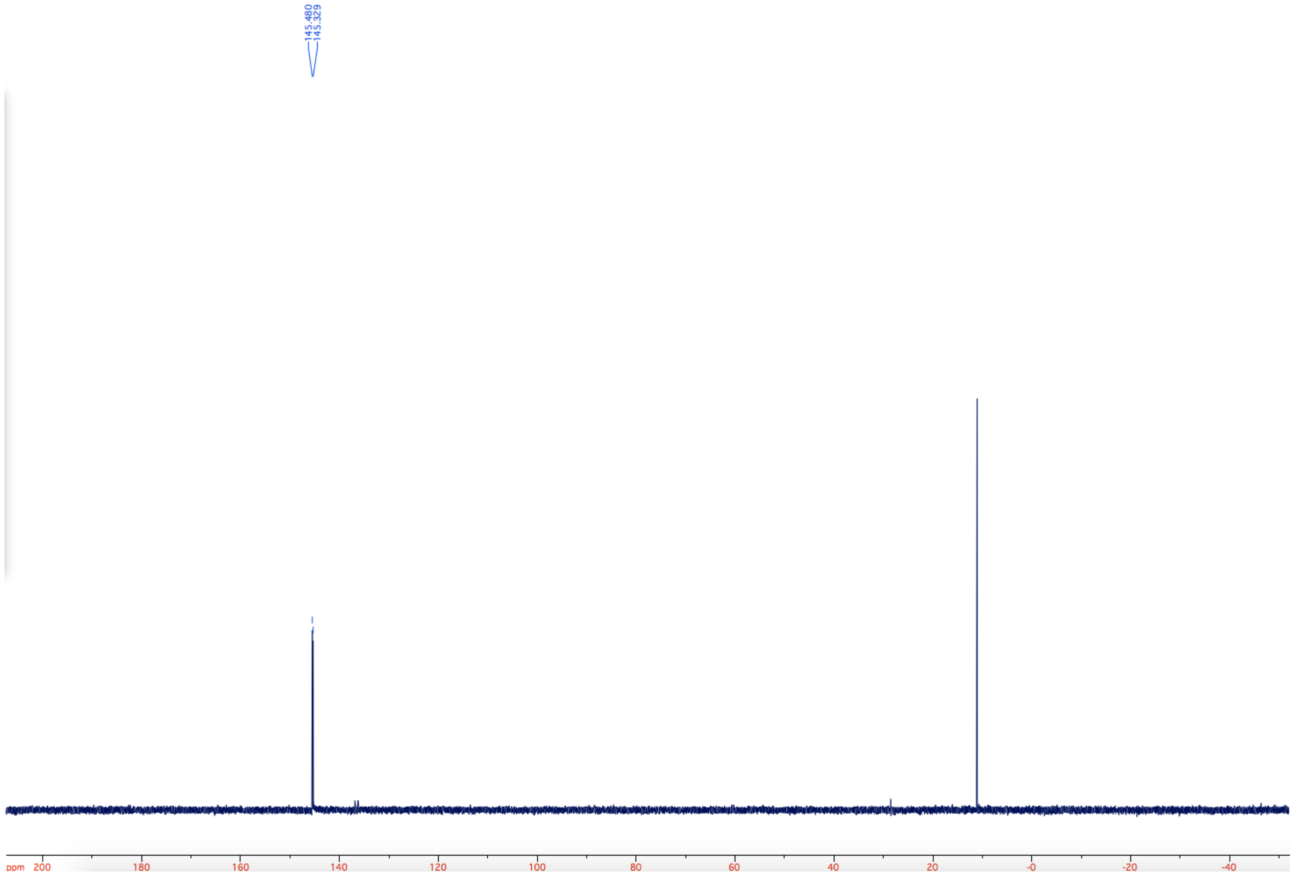

Figure S10. ^{31}P NMR spectrum of compound **2**. The signal at 11 ppm comes from an impurity.

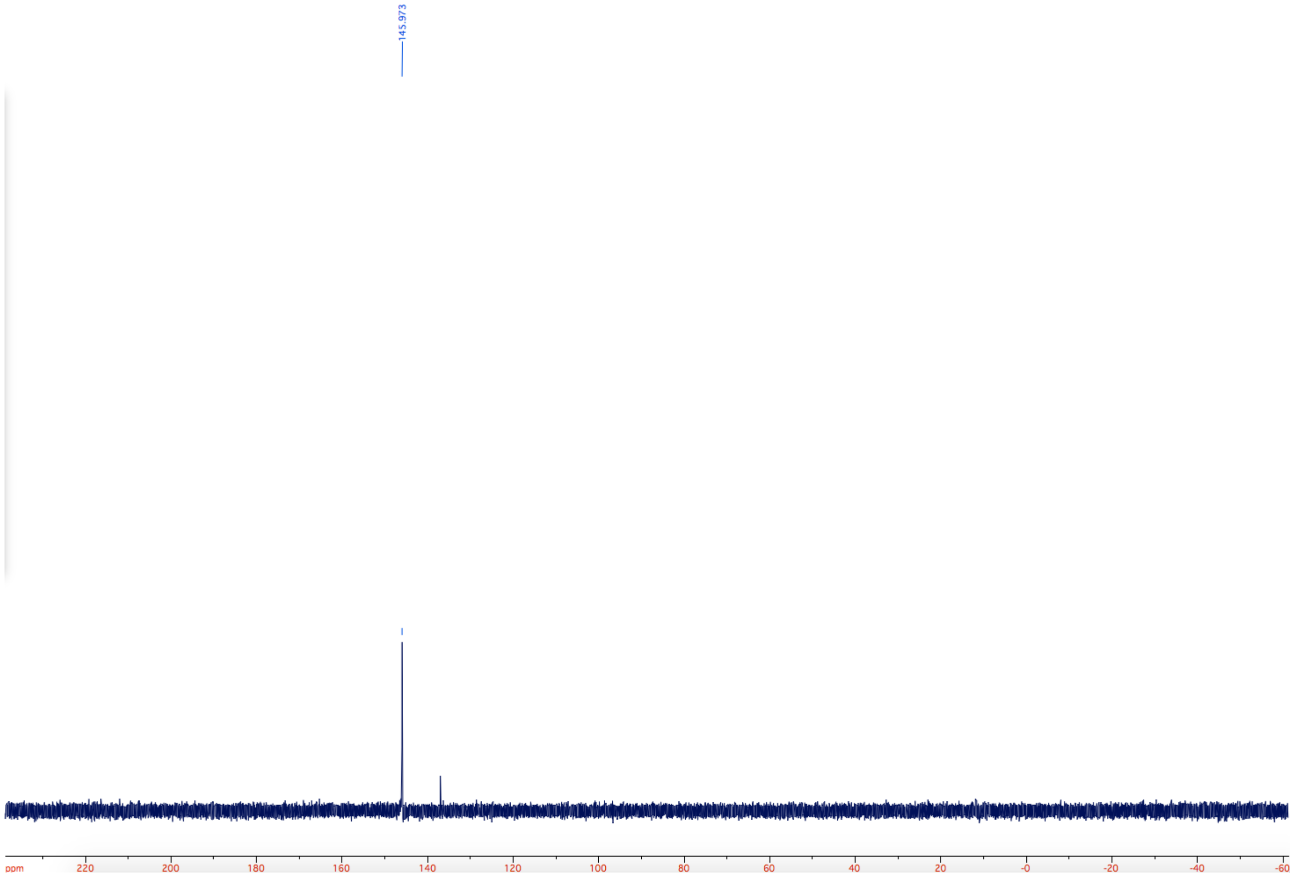

Figure S11. ^{31}P NMR spectrum of compound **3**.

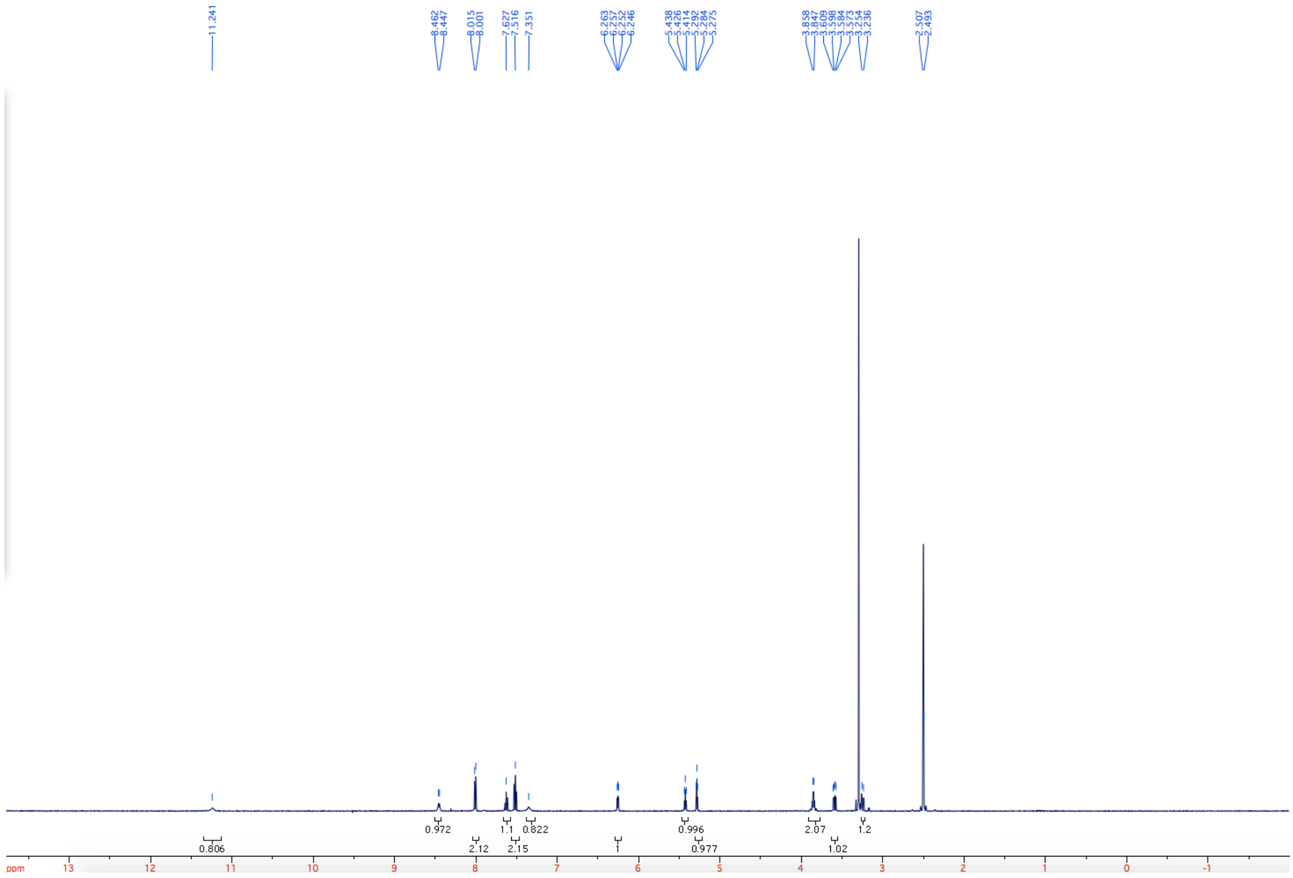

Figure S12. ^{1}H NMR spectrum of compound **10**.

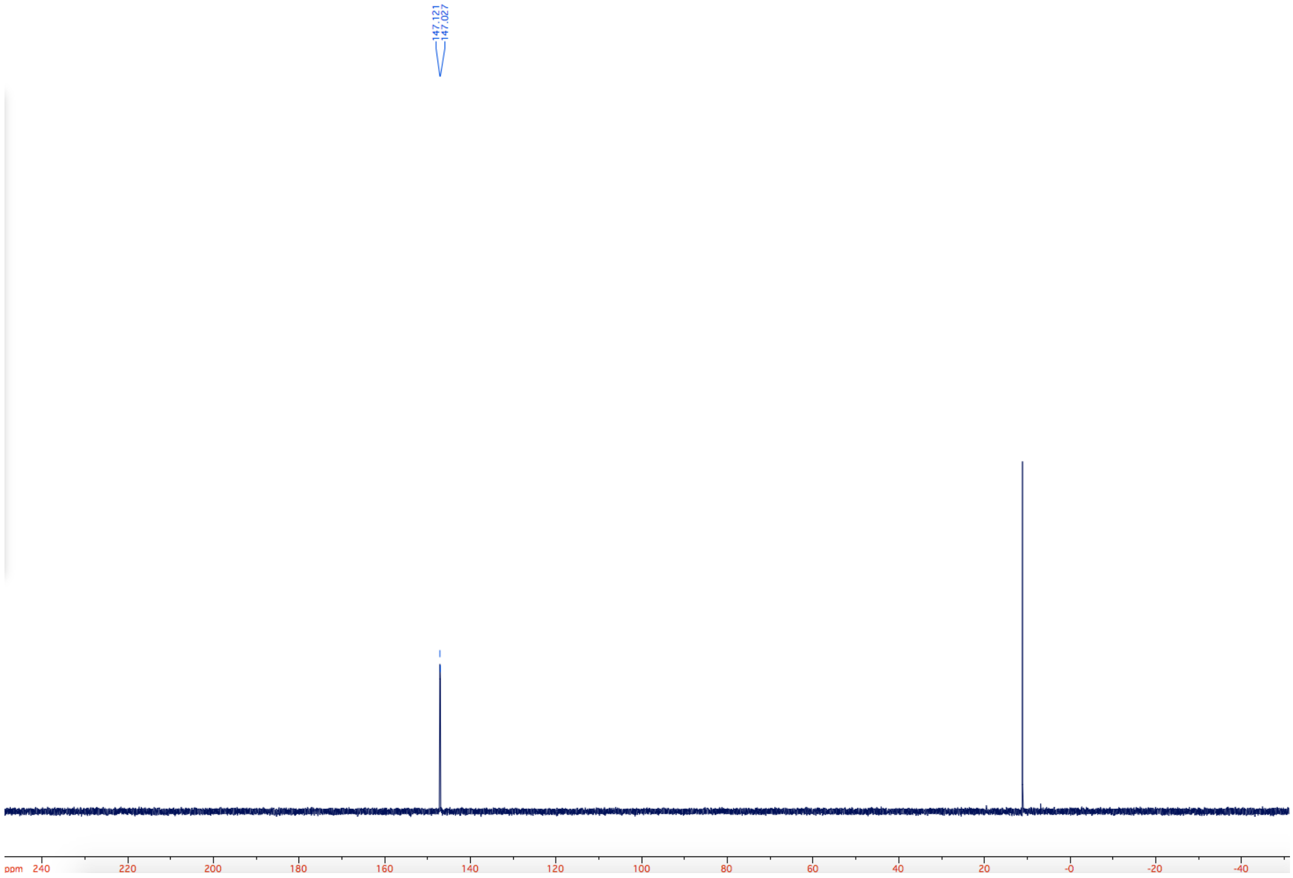

Figure S13. ^{31}P NMR spectrum of compound **4**. The signal at 11 ppm comes from an impurity.
